# Transcriptomic analysis of the spatiotemporal axis of oogenesis and fertilization in *C. elegans*

**DOI:** 10.3389/fcell.2024.1436975

**Published:** 2024-08-19

**Authors:** Yangqi Su, Jonathan Shea, Darla Destephanis, Zhengchang Su

**Affiliations:** Department of Bioinformatics and Genomics, The University of North Carolina at Charlotte, Charlotte, NC, United States

**Keywords:** *C. elegans*, oocyte, oogenesis, transcriptome, single cell, ScRNA-seq

## Abstract

*Caenorhabditis elegans* hermaphrodite presents a unique model to study the formation of oocytes. However, the size of the model animal and difficulties in retrieval of specific stages of the germline have obviated closer systematic studies of this process throughout the years. Here, we present a transcriptomic level analysis into the oogenesis of *C. elegans* hermaphrodites. We dissected a hermaphrodite gonad into seven sections corresponding to the mitotic distal region, the pachytene region, the diplotene region, the early diakinesis region and the 3 most proximal oocytes, and deeply sequenced the transcriptome of each of them along with that of the fertilized egg using a single-cell RNA-seq (scRNA-seq) protocol. We identified specific gene expression events as well as gene splicing events in finer detail along the gonad and provided novel insights into underlying mechanisms of the oogenesis process. Furthermore, through careful review of relevant research literature coupled with patterns observed in our analysis, we delineate transcripts that may serve functions in the interactions between the germline and cells of the somatic gonad. These results expand our knowledge of the transcriptomic space of the *C. elegans* germline and lay a foundation on which future studies of the germline can be based upon.

## Introduction

With a transparent body of less than 1,000 somatic cells, a fully sequenced genome harboring 19,985 protein-coding genes (based on the WS291 annotation) and about 14 h of embryogenesis time and 2 weeks of life span, the *C. elegans* hermaphrodite provides an extraordinary model to understand cell differentiation and organogenesis (Sulston and Horvitz, 1977; Sulston et al., 1983; Wood and Edgar, 1994; Consortium, 1998; Rose and Kemphues, 1998; Labouesse and Mango, 1999; Hillier et al., 2005; Kim et al., 2013a; Chu and Shakes, 2013; Marcello et al., 2013; Robertson and Lin, 2013). Particularly, *C. elegans* gonad provides an excellent model to understand meiosis (Pazdernik and Schedl, 2013), gamete formation (Kim et al., 2013a; Chu and Shakes, 2013) and fertilization (Marcello et al., 2013).

**FIGURE 1:**
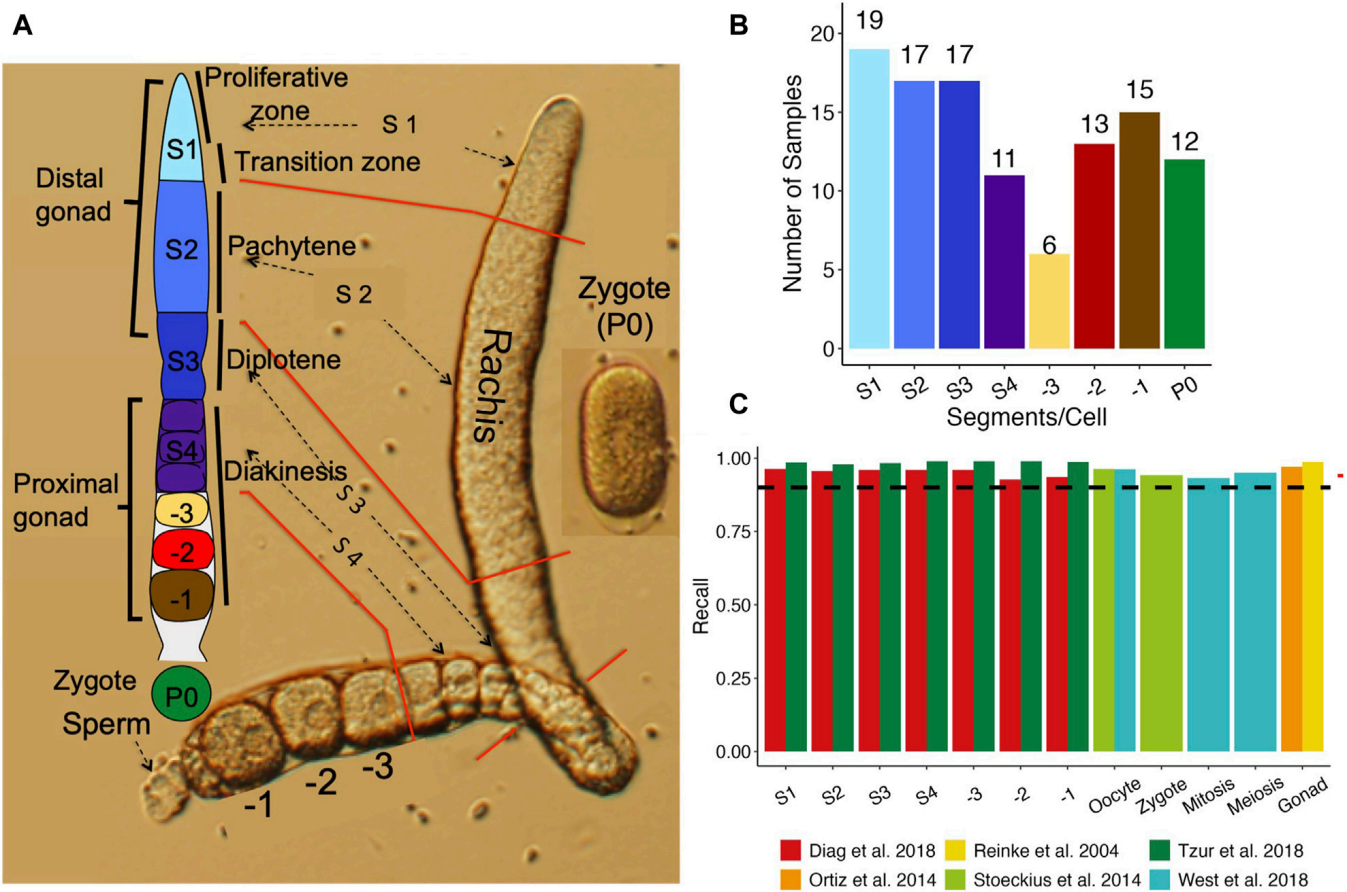
Comparison of our datasets with existing ones. **(A)** A diagram of an isolated one side gonad together with a cartoon of one side gonad showing the dissection positions for the segments along the one side gonad. **(B)** Number of samples from each stage of segments, oocytes and zygotes. **(C)** Percentage of genes found expressed in each stage in previous studies that we found expressed in our study.

In the *C. elegans* hermaphrodite germline, oogenesis occurs independently in two sets of U-shaped gonads connected to a single shared uterus (Pazdernik and Schedl, 2013). Oocyte formation begins at the distal end of each gonad with mitotically proliferating germline stem cells near the single somatic distal tip cell (DTC) (Kimble and White, 1981). Proliferating germ cells moving away from the DTC and begin to enter meiosis prophase I through a transition zone, after which germ cells move along the gonad while going through the pachytene, diplotene and diakinesis stages, and ending in the most proximal (-1) oocytes that awaits fertilization in the spermathecae for progression into metaphase I and the subsequent formation of the zygote (Kim et al., 2013b). Apart from the proximal oocytes in diakinesis, most of the germline nuclei do not have fully enclosed membranes and form a syncytium, sharing a nucleus free cytoplasmic region called the rachis, which facilitates the transport of RNAs and proteins to growing oocytes (Figure 1A) (Wolke et al., 2007; Nadarajan et al., 2009). Throughout this process, the germline also is enveloped by five pairs of gonadal sheath cells (Sh1-Sh5 from distal to proximal), each pair serving distinct functions through communication with the germline and promoting the oogenesis program (Hall et al., 1999; Killian and Hubbard, 2005).

However, for a long time this system is limited by its miniscule size, preventing a detailed dissection of the biochemistry in each part of the oocyte assembly line using techniques such as transcriptome profiling using microarray (Reinke, 2002; Walhout et al., 2002; Baugh et al., 2003) or bulk-RNA sequencing (RNA-seq) (Gerstein et al., 2010; Spencer et al., 2011; Li et al., 2014), and proteome profiling using mass spectrometry (Yuet et al., 2015), as all of these techniques require a descent quantity of RNA/protein from at least hundreds of thousand cells.

Recent studies have performed micro-dissections of the *C. elegans* gonad and profiled transcriptomes of dissected segments using single-cell RNA-seq (scRNA-seq) techniques (Diag et al., 2018). However, these analyses mainly focused on the post-transcriptional/translational regulation of germline transcripts via binding of 3′UTRs to RBPs and miRNAs. Although these studies provided expression estimates for genes from each segment as well, they did not focus on other aspects of the transcriptome between the segments that might also account for the progress of oogenesis. Consequently, the research community still lacks a good understanding of the machinery of the assembly line, such as key regulators and gene expression patterns along the temporal and spatial axis of the gonad.

To fill these gaps, we combined microdissection with scRNA-seq technique (Tang et al., 2010a; Tang et al., 2010b; Ramskold et al., 2012; Picelli et al., 2013), and profiled the transcriptomes in the proliferative zone, pachytene zone, diplotene zone, early diakinesis zone (before -3 oocyte stage), later diakinesis zone (-3, -2, -1 oocytes), and the zygote. Our results revealed a highly dynamic picture of gene transcriptional regulation at each transitional time point throughout the oocyte assembly line. These results should provide a foundation to further investigate the molecular mechanisms of the oogenesis and fertilization processes.

## Materials and methods

### Experimental model

The AZ212 *C. elegans* strain (pie-1::H2B::GFP) was obtained from the *C. elegans* Genetics Center (University of Minnesota), and was maintained in *E. coli* OP50 lawn on an agar plate according to the standard protocol (Stiernagle, 2006).

## Method details

### Dissection of the gonad and harvest of samples

After a well-fed gravid hermaphrodite was immobilized in the egg salt solution (ESS) containing 118 mM NaCl and 48 mM KCl (Edgar and Goldstein, 2012) with 10% tetramisole (Sigma, St. Louis), a cut was made across the vulva using a 26G subcutaneous needle controlled by a micromanipulator (ROE-200, Sutter) under an inverted microscope (Olympus 1X71). This would release fertilized eggs and early-stage embryos from the uterus as well as sperm and at least portions of the two sides of the gonad. Each end of the gonad wrapped around by five pairs of sheath cells was completely isolated by pushing its distal end as shown in Figure 1A. The -1, -2 and -3 oocytes as well as the diakinesis zone (S4), the loop corresponding to the diplotene zone (S3), the pachytene zone (S2), and the distal proliferative zone (S1) were sequentially isolated by a cut at the positions as shown in Figure 1A, and similarly harvested. The zygote (fertilized oocyte) also known as P0 was similarly harvested when the two pronuclei were fused at its center (Figure 1A). Once a sample was harvested, it was immediately transferred to a 200 μL PCA tube containing 4 μL cell lysis buffer (0.45 μL 10X PCR buffer II, 3 mM MgCl_2_, 0.45% NP40, 4.5 mM DTT, 0.18U/μL SUPERase-In, 0.36U/μL RNase inhibitor, and 2 mM dNTP. All the reagents in the buffer were from Life Technologies except NP40 (Roche). The PCR tube containing the sample was incubated in a thermocycler at 70°C for 90 s and then transferred on ice before being stored at −80 until use. Due to the difficulty for their isolation, sheath cells wrapped around the gonad segments and oocytes were also harvested in the samples. Moreover, despite meticulous care taken during sample collection, some samples may contain sperm released during gonad dissection. In total, we harvested 136 samples, including 24 S1 segments, 24 S2 segments, 20 S3 segments, 16 S4 segments, 7 -3 oocytes, 15 -2 oocytes, 19 -1 oocytes, and 11 zygotes (P0).

### Preparation of RNA-seq libraries

In the early stages of the project, we prepare a sequencing library for each harvested sample for Illumina platforms using a modified scRNA-seq method based on Tang et al. as previously described (Tang et al., 2010a; Tang et al., 2010b; Su et al., 2023). Samples prepared during this time were either sequenced by 100 bp single end reads on an Illumina HiSeq2000 with an average of 37, 508, 654 reads/sample or were sequenced by 100 bp paired-end reads on an HiSeq2500 machine with an average of 17, 468, 703 reads/sample). Later on, we prepared samples using the Smart-seq2 protocol (Picelli et al., 2013) later on. Samples produced during this stage constitute most of our samples, which were sequenced by 125 bp pair-end reads on an Illumina HiSeq2500 machine with an average 3,575,998 reads/sample.

### Transcriptome mapping and quantification

The *C. elegans* genome assembly (GCA_000002985.3) was obtained from NCBI Refseq, while the annotations were based on Wormbase version: WS291. Prior to mapping, raw reads were trimmed with Trim Galore (Krueger, 2015), with parameters (quality ≥10, length >35 bp). We quantified the expression levels of genes in two ways for different subsequent analysis. For differential gene expression analysis, trimmed reads were mapped to the genome using HISAT2 (Kim et al., 2019) with default settings, read counts were obtained by using HTSeq (Anders et al., 2015) with default settings based on the mapping results. The trimmed reads were also mapped to the genome using Salmon (Patro et al., 2017) with default settings to obtain transcript per million (TPM) estimates for both genes and transcripts.

### Quality control

Sequenced libraries were then assessed for quality with custom scripts and quality metrics evaluated via the QoRTs package (Hartley and Mullikin, 2015). First, we designated certain genes as mitochondrial, ribosomal, sperm associated, intestine associated, or stress associated based previous publications. Specifically, ribosomal genes and mitochondrial genes were selected based on gene annotations (Davis et al., 2022). Selection of sperm, intestine and stress associated genes were based on manual curation of genes from earlier studies along with a correlation analysis of gene expression. Briefly, sperm associated genes are a combination of curated sperm genes from previous studies (Reinke et al., 2004; Ortiz et al., 2014) and genes whose expression levels were highly correlated with those of major sperm protein genes (Supplementary Figure S1; Supplementary Table S1). Similarly, intestine associated genes were a combination of curated intestine genes in a previous study (Mcghee, 2007) and in WormBase (Davis et al., 2022) and genes whose expression levels were highly correlated with those of the curated intestine genes (see specifics in Supplementary Figure S1; Supplementary Table S1). Finally, stress related genes are a commination curated stress genes in a previous study (Brunquell et al., 2016) and genes whose expression levels are highly correlated with the curated stress genes (Supplementary Figure S1; Supplementary Table S1). A sample was filtered out if it met any of the following criteria: i) over 5% reads (in terms of TPM) were from the mitochondrial genome; ii) over 5% reads (in terms of TPM) were from rRNA genes; iii) over 5% reads (in terms of TPM) were from sperm specific genes); iv) over 5% reads (in terms of TPM) were from intestine specific genes; v) HISAT unique reads mapping rate <70%; vi) less than 50% of HISAT uniquely mapped reads were mapped to coding DNA sequences. These criteria were set to remove samples that were of poor libraries quality or were heavily contaminated by sperm, intestinal tissue and/or exhibited reduced quality during sample collection. To further increase the robustness of subsequent analysis, samples were visualized using Uniform Manifold Approximation and Projection (UMAP), and those that largely deviated from clustered groups of the same sample type were removed. We also included the 6 P0 (1-cell) samples of Tintori et al. (2016) in our analysis, and the samples were processed through the same pipeline as our own samples. We note that with exception of comparisons with previously published datasets, all our subsequent analyses were performed with genes excluding all mitochondrial, ribosomal, stress, sperm and intestine associated genes.

### Comparison with previous datasets

Gene expression data from six previous studies were collected from the following sources and compared with our data. For all comparisons, we used filtered samples with all genes (genes were not filtered). Details of the datasets and comparisons are as follows:

1) Reinke et al. (2004) provided the first microarray-based list of oogenic genes. The list was retrieved from via their Supplementary Material.
2) Ortiz et al. (2014) performed RNA-seq analysis on the gonad to distill a list of genes termed oogenic. These genes were acquired via their Supplementary Material, and genes marked oogenic were used for our subsequent comparisons.
3) Stoeckius et al. (2014) performed RNA-seq on proximal oocytes and 1 cell zygotes. Expression profiles were acquired via the instructions in their paper and genes with expression >0.5 RPKM were deemed expressed.
4) West et al. (2018) dissected the gonad into mitotic and meiotic sections, and oocytes. RNA-seq data of each sample was acquired via the Supplementary Material of the paper, and genes with a reads count >0 were deemed expressed.
5) Tzur et al. (2018) utilized the Cel-seq protocol to sequence 10 segments of the *C. elegans* gonad, with 2 replicates per segment. Alignment of these 10 segments to our segments was based on diagrams presented in their study and rough estimates of where their dissection occurred. The exact alignments between their segments and ours are given in Supplementary Table S2. Count matrices were acquired per the authors’ instructions. Pearson correlation was performed with log transformed count values using all shared genes.
6) Diag et al. (2018) performed cryo-dissection of the 3 posterior and 3 anterior gonads into 13–15 segments per gonad. This resulted in 85 slices sequenced via Cel-seq. Expression profiles for these samples were retrieved from GEO with accession number GSE115884. Samples with <10^4^ reads were discarded from correlation analysis with our samples. The authors (Diag et al., 2018) provided approximate slice label, slice size as well estimates size of each gonad region. Thus, we were able to derive a coarse conversion from their slices to our segments, as shown in Supplementary Table S2. Pearson correlation was performed with log transformed count values using all shared genes.

### Differential gene expression analysis

We performed differential gene expression analysis between each two dissected neighboring stages along the developmental axis of the gonads as above-described and zygotes using Monocle2 (Qiu et al., 2017). Experimental batch and gene detection rate in each sample were included as covariates along with segment/cell-type to model normalized gene expression using the negbinomial. size model of Monocle2. Because Monocle2 does not produce Log_2_FoldChange (Log_2_FC) values, we applied Bayesian shrinkage of gene model coefficients using the apeglm (Zhu et al., 2019) package to account for large foldchange values of genes with low expression and obtain shrunken Log_2_FC values for each gene. A model of gene expression as a function of segment/cell-type was also fit to assess genes that were differentially expressed across all stages prior to fertilization (excluding P0). Genes with an Benjamini-Hochberg adjusted *p*-value (BH p-adj) <0.05 and a fold change increase/decrease of 1.5 were considered differentially expressed. ClusterProfiler (Yu et al., 2012) was used to perform Gene Set Enrichment Analysis (GSEA) with pre-ranked shrunken Log2FC values and gene sets from KEGG (Kanehisa et al., 2022), GO (Gene, 2021) Biological Pathways, Reactome (Milacic et al., 2024) and Wikipathways (Martens et al., 2021). Enrichment of each type of gene sets was performed separately, and the results were aggregated. Only gene sets containing more than 10 and less than 250 genes were considered, and those with a q value <0.05 were considered significantly enriched.

### Clustering Co-expressed genes

The union of DEGs identified in all pairwise comparisons were used for gene co-expression analysis. After the read count values of genes were variance stabilizing transformed using the vstExprs function of Monocle2 package, Pearson correlation coefficient between expression levels of the genes in the samples were calculated, and genes were hierarchically clustered using the “ward.D2” method of the hclust function in R. Upon visual inspection of the resulting clustering heatmaps, the clusters were set at a hierarchical level. Each cluster was then subject to enrichment analysis for GO biological process (BP) terms using ClusterProfiler (Yu et al., 2012) to identify significantly enriched terms for the cluster. Gene expression as well as the respective clusters were visualized with the ComplexHeatmap package (Gu, 2022), and the top three most significantly (q value < 0.05 or *p*-value < 0.001) enriched GO terms were shown alongside the heatmap.

### Validation of expression patterns of DEGs using *in situ* hybridization (ISH) images in the NEXTDB database

For an identified DEG cluster, we focused only on genes that have *in situ* images in NEXTDB database (Shin-i and Kohara, 1999). We then selected genes that had an average normalized expression level >500 across all gonadal segments (S1 to -1, we did not include zygote expression due to inconsistent staining of zygotes in the NEXTDB database) to ensure visible imaging signals in at least one segment for most genes. We denote the maximum Fold Change (FC) for a gene as the maximum absolute FC of the gene across all its FCs between adjacent gonad segments (S1 vs. S2, S2 vs. S3, etc.). We then select at most 10 genes with highest maximum FC values. If the cluster had less than 5 genes selected, we then considered all genes with an average expression level >100 in at least one of the segments and selected at most 5 genes with highest maximum FC values. For each selected gene, we manually examined the ISH images of its transcripts in NEXTDB.

### Differential alternative polyadenylation analysis

3′UTR regions were extracted from the WS291 annotation via custom scripts to only include 3′UTR regions that did not overlap coding exons and other UTR regions. The Samtools (Li et al., 2009) depth function was used to obtain pair-read aware coverage of the genome for each samples with HISAT2 (Kim et al., 2019) aligned bam files. Coverage for each sample was normalized with DESeq2 (Love et al., 2014) size factors before estimation of polyadenylation site and long/short 3′UTR coverage, and Percentage of Distal poly-A site Usage Index (PDUI) was computed performed using DaPars2 (Feng et al., 2018; Li et al., 2021). Modification to the DaPars2 program was made to begin polyadenylation site search starting from 25 bp downstream of 3’UTR’s 5′ end. For each neighboring stages comparison, only 3′UTRs that belonged to a gene with a mean count >10 across all compared samples and had PDUI values in at least three samples in both stages were tested for differential alternative polyadenylation. Fisher’s exact test was performed with the average long/short 3′UTR coverage in compared stages, and the resulting *p*-values were corrected for false discovery rate (FDR) via the Benjamini Hochberg method. Genes that had FDR <0.05 and |PDUI difference| >0.05 were called for significantly differential alternative polyadenylation. ClusterProfiler (Yu et al., 2012) was used to perform GO BP (Ashburner et al., 2000) term enrichment analysis, and significant terms with FDR <0.05 were called significantly enriched. Visualization was made with the trackViewer (Ou and Zhu, 2019) R package.

### Differential splicing analysis

Differential splicing analysis was performed using rMATS (Shen et al., 2014) that calculated splicing Psi values and evaluated their statistical significance. rMATS classifies splicing events into five categories: alternative 3′ splice site (A3SS), alternative 5′ splice site (A5SS), retained intron (RI), mixed exon usage (MXE), skipped exon usage (SE). A splicing event with a Psi value change >0.1 and an adjusted *p*-value <0.05 was significant.

## Results

### Expression levels of detected genes correlate well with those from previous studies

We cut each isolated gonad into seven segments roughly corresponding to the stages of oocyte development (Figure 1A) (Materials and Methods), and the number of samples collected for each segment, oocyte and the zygote are shown in Figure 1B. To assess the quality of the RNA-seq libraries, we evaluated the similarity between the detected genes and their expression values and those from six previous studies (Reinke et al., 2004; Ortiz et al., 2014; Stoeckius et al., 2014; Diag et al., 2018; Tzur et al., 2018; West et al., 2018) (Materials and Methods). Four (Reinke et al., 2004; Ortiz et al., 2014; Stoeckius et al., 2014; West et al., 2018) of these studies largely quantified expression levels in entire gonads or large sections of the gonad, thus we aggregated gene expression in corresponding samples to allow reasonable comparisons. Our aggregated expression profiles recall over 90% of expressed genes in all the four datasets (99% for Reinke et al., 2004; 93% for Ortiz et al., 2014; 95% for Stoeckius et al., 2014; 96% for West et al., 2018) (Figure 1C), indicating that our data are consistent with these earlier results.

Furthermore, two of these studies (Diag et al., 2018; Tzur et al., 2018) dissected the *C. elegans* gonad into multiple segments and profiled the transcriptome of each segment using a variety of techniques including RNA-seq. As both studies cut the gonad in more segments than we did, we thus aggregated data from the segments of (Diag et al., 2018) and (Tzur et al., 2018) according to the alignments of the segments (Materials and Methods; Supplementary Table S2), so that data from largely the same segments as ours were compared. Our detected genes in each segment/oocyte recall most of detected genes in the corresponding aggregated segments by (Tzur et al., 2018) and (Diag et al., 2018) (Figure 1C). Moreover, the expression levels of genes in our segments are largely correlated with those in the corresponding aggregated segments in the two prior studies (Supplementary Figuers S2A, B). These results suggest that we have largely correctly align the segments in both studies to ours. However, notably, our detected genes have higher recall rates (Figure 1C) for and higher correlation coefficients (Supplementary Figuers S2A, B) with those of (Tzur et al., 2018) than for and with those of (Diag et al., 2018). This might be due to the higher similarity in gonad dissection between our segments and those of (Tzur et al., 2018) than between our segments and those of (Diag et al., 2018). These results further suggest that our detected genes are largely consistent with those detected by previous studies.

### Differential gene expression mostly occurs in early stages of oogenesis and -1 proximal oocytes

Dissection of the *C. elegans* hermaphrodite gonad is a delicate procedure that is prone to contamination from neighboring tissues, particularly, intestine cells and released sperm. To mitigate the effects of such contaminations, we filtered sperm-, intestine- and stress-related genes in the samples and discarded heavily contaminated samples following a procedure (Materials and Methods; Supplementary Figuers S1; Supplementary Table S1).

**FIGURE 2:**
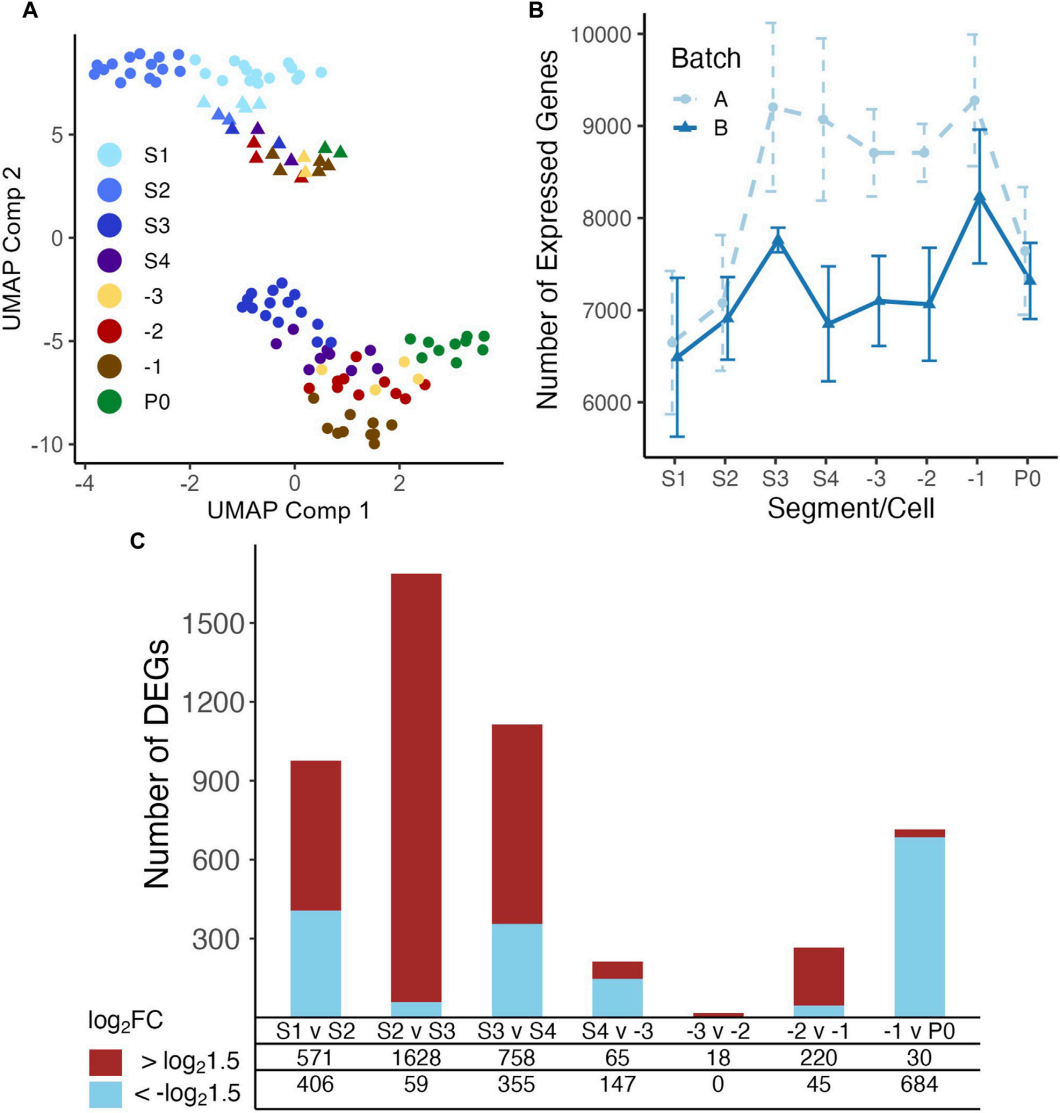
UMAP display of samples and differential expression analysis of genes between neighboring stages. **(A)** Both batches of samples are clustered according to their positions along the gonad developmental axis by UMAP based on their measured transcriptomes. Libraries of batch A were mainly prepared using a modified Tang protocol (Tang et al., 2010a; Tang et al., 2010b; Su et al., 2023), and libraries of batch B were mainly prepared using the Smart-seq2 protocol (Picelli et al., 2013). **(B)** Boxplot of numbers of genes detected in the samples in each developmental stage of the gonad and zygotes. **(C)** Number of upregulated and downregulated genes detected between each pair of neighboring stages, see Supplementary Table S3 for details.

We inspected the relationships among our samples via UMAP visualizations. As shown in (Figure 2A), the samples form into two distinct clusters, indicating strong batch effects in our datasets possibly due to the two different scRNA-seq library preparation protocols used at different stages of the project (Materials and Methods). Nonetheless, a trajectory from S1 samples to -1 oocytes and zygote samples is formed in both batches, which is in line with the developmental path of the germline. Thus, we account for batch effects in subsequent analysis when possible. Inspection of the number of genes expressed in each segment/cell type shows a clear pattern, i.e., the number of expressed genes increased from S1 to S3, before dropping slightly in S4 and exhibiting only minor changes before another increase in the -1 oocyte and finally a large decrease in the fertilized oocyte (Figure 2B). Therefore, it appears that gene transcriptional regulation mostly occurs in early stages of oogenesis, particularly between the S2 (pachytene) and S3 (diplotene) transition and becomes progressively quieter as the oocyte goes through the S4 stage and -3/-2 oocytes (Figure 2B). Gene transcription appears to reactivate in the -1 oocyte as a potential preparation for fertilization (Figure 2B). To further reveal gene expression transitions along the developmental axis of the gonad, we analyzed DEGs between each pair of neighboring stages with the earlier stage as the baseline reference in each comparison (Figure 2C; Supplementary Table S3). Transition from S2 to S3 invokes the largest number of upregulated DEGs, and transition from -3 to -2 has the smallest number of DEGs, while fertilization triggers the largest number of downregulated DEGs in the zygotes (Figure 2C).

### DEGs form distinct clusters that are significantly enriched for various functions related to oogenesis

**FIGURE 3:**
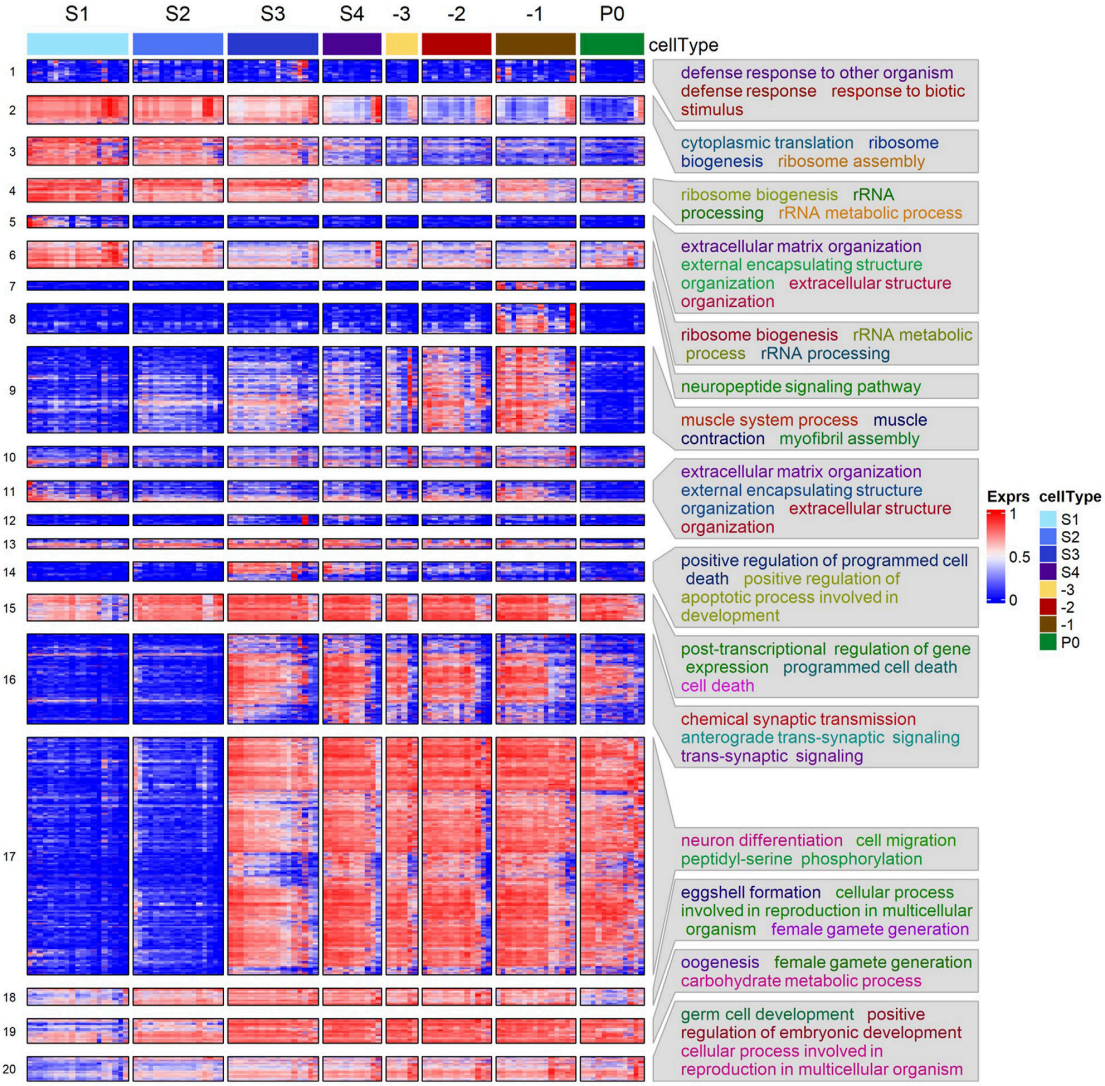
Heatmap of hierarchical clustering of DEGs using their transcription levels across the seven stages of oogenesis and zygotes. Enriched GO BP terms in some clusters are shown. See Supplementary Table S4 for details.

To reveal functional modules underlying the maturation process and fertilization of oocytes, we clustered the union of DEGs identified in all neighboring stages comparisons, based on their expression levels in all analyzed samples. As shown in Figure 3, the DEGs form 20 distinct clusters that are significantly enriched for various functional modules. For instance, clusters 2, 4 and 6 are significantly enriched for ribosomal and translation related processes. All these three clusters of genes exhibited a downregulating trend of expression, albeit with their largest decrease at different stages. Cluster 14 and 15 are enriched for genes involved in programmed cell death, with expression levels elevated in the S3 stage corresponding to the diplotene loop. However, genes in cluster 14 were quickly downregulated after the S4 stage, while genes in cluster 15 retained similar transcription levels through the subsequent stages. Cluster 18 -20 are all enriched for processes related to oogenesis, e.g., eggshell formation and female gamete generation. Genes in these three clusters exhibited increasing trends of expression from S1 to -1 the most proximal oocyte (-1), with the largest increases happening in the early stages (S1-S3). However, genes in cluster 18 experienced reduced expression after fertilization in the zygotes (P0 cells), while genes in cluster 19 and 20 remained at similar expression levels. Furthermore, genes in cluster 18 are enriched for eggshell formation, suggesting that transcripts-related to eggshell formation begin degradation post-fertilization after their protein products are no longer needed. Most DEGs belonging to the larger clusters 16 and 17 exhibited similar increases in expression from S2 to S3 and maintained steady levels of expression throughout the later stages even post fertilization. These genes are involved in phosphorylation, synaptic transmission and signaling, positive regulation of transcription, neuronal differentiation, cell fate specification and cell migration. Interestingly, cluster 17 is strongly enriched for genes involved in neuronal development, suggesting common functional modules might be used in the differentiation processes of both neurons and oocytes. Cluster 9 is enriched for genes involved in muscle structures and myofibril assembly. As mentioned above, proximal gonadal sheath cells serve the role of pushing oocytes into the spermathecae and require many components like those of muscle cells. Thus, it is highly likely that genes of this cluster originate from proximal sheath cells wrapped around the proximal oocytes. It is also worth noting that gene expression pattern of cluster 9 differ from those of clusters 16 and 17 in that expression of genes in cluster 9 almost completely disappears in fertilized zygotes, likely due to the absence of sheath cells surrounding the isolated zygotes. Cluster 1 exhibits no obvious pattern of change in expression and the expression levels are generally low. These genes are enriched for defense response related processes and might be required at low levels along the gonad temporospatial axis. Both clusters 5 and 11 are enriched for extracellular matrix organization. It has been shown that many genes (*mig-6*, *mig-39*, *lag-2*, *let-2*, *epi-1*, etc.) in the two clusters (Supplementary Table S4) were preferentially expressed in the distal mitotic regions of the gonad and played roles in extracellular matrix organization and distal tip cell migration (Henderson et al., 1994; Huang et al., 2003; Kawano et al., 2009; Kikuchi et al., 2015). Consistently, expression levels of these genes were elevated in S1.

We also performed GSEA using shrunken log2FC values of all genes evaluated between each pair of neighboring stages and the results are summarized in Supplementary Table S4. Although most of the GSEA results are in accordance with those observed in the gene clustering enrichments (Figure 3), surprisingly, GSEA finds upregulated genes enriched for cell cycle activity, mitosis, transcription, mRNA splicing, mitochondrial translation, and ATP production in the -1 oocytes vs. P0 comparison (Supplementary Table S5). This suggests that transcriptional activation of cell division and energy production is present in the zygote.

### Detected gene expression patterns mostly align with ISH images in the NEXTDB database

To validate our detected gene expression patterns along the developmental axis of the gonad, we resorted to ISH images in the NEXTDB database (Shin-i and Kohara, 1999). Of our 3,520 DEGs, 2,223 have ISH images in NEXTDB. However, not all *in situ* images showed clear imaging of the L4 stage worm gonad. Furthermore, genes with low expression, very high expression or relatively low FC along the gonad segments will likely exhibit poor clarity of expression pattern. Thus, to validate our expression results, we attempted to select for genes that would likely exhibit clear expression patterns in NEXTDB for each of the gene clusters, except for cluster 1 and 8. We did not validate genes in cluster 1 due to the enrichment of defense to pathogen, suggesting genes of cluster 1 were likely due to stress in some segments/cells, and may not represent interesting patterns that underlie oogenesis, while validation of genes in cluster 8 is shown separately in subsequent sections.

In total, we selected 121 DEGs that were distributed in 17 of the 20 clusters (Supplementary Table S6), no DEGs in cluster 12 matched our selection criteria. Of these 121 DEGs, 80 genes (67%) distributed in 14 clusters have clear staining of the gonad in NEXTDB. Examples of images of 44 of these genes along with our detected expression pattern are shown in Supplementary Figures S13–S15. Our detected expression patterns of the genes along the gonad are in excellent agreement with the corresponding images. For instance, in the case of the *mig-6* gene of cluster 5, ISH images show that its expression is only evident in distal tip cells, which is reflected in our diagram of gene expression pattern showing its decreased expression from S1 to S2. Other examples such as that of ribosomal protein subunit genes *rps-21*, *rps-22* and *rpl-11*.*1* show clear decreasing ISH staining in accordance with our expression results (Supplementary Figure S3). Genes increasing in expression during oogenesis such as that of *cpg-1/2* and *egg-1* also show accordance with their respective ISH Supplementary Figure S13). We further note that genes from cluster 9, which was enriched for muscle related genes, show staining around proximal oocytes, reinforcing our hypothesis that genes of cluster 9 may be mainly of sheath cell origin (Supplementary Figure S7). We thus conclude that our detected gene expression patterns along the gonad are generally accurate.

### *In silico* analysis of proximal oocytes and zygotes may uncover putative sheath cell expression

**FIGURE 4:**
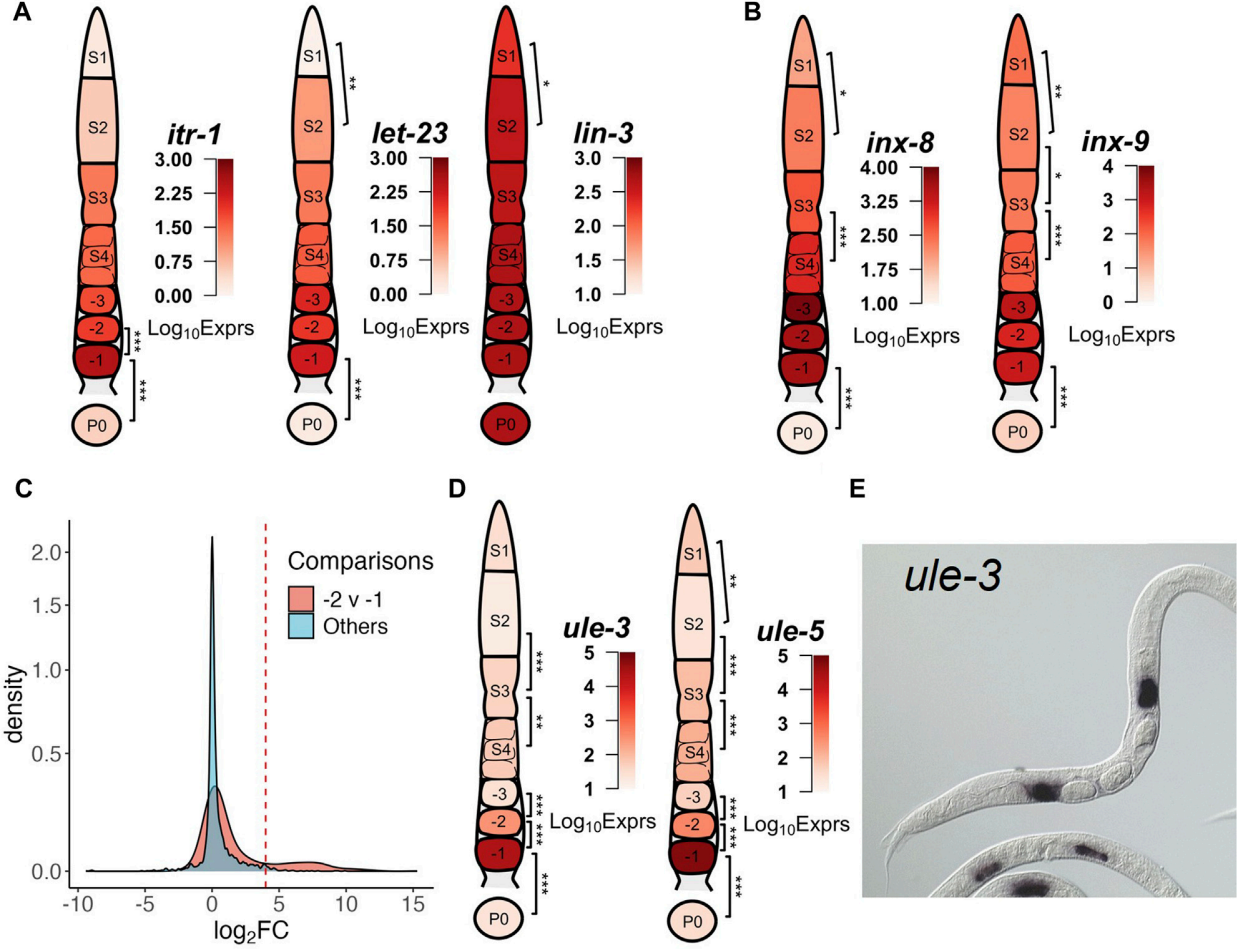
Examples of transcriptional dynamics of possible sheath cell genes along the gonad developmental axis. **(A)** Genes coding for hemichannels (*inx*-8 and *inx*-9) of the somatic gonad. **(B)** Genes coding for components signaling pathways between proximal sheath cells and oocytes. **(C)** Distribution of Log_2_FC values between neighboring stages of the DEGs that are significantly downregulated in the −1 vs. P0 comparison. A small portion of these DEGs is significantly upregulated in the −2 vs. −1 comparison as indicated by the right peak of the distribution compared to other comparisons, see Supplementary Table S7 for details. **(D)** Genes coding for ULE-3/5. **(E)** NEXTDB *in situ* imaging of ule-3 expression in spermathecae. In each gonad diagram, the average expression levels of the genes in each segment or the zygote are shown. BH p-adj: * <0.05; ** <0.01; *** <0.0001.

The hermaphrodite gonad is tightly wrapped by five pairs of sheath cells that provide germline maturation signals, move germ cells along the rachis and push proximal oocytes into the spermathecae (Hall et al., 1999; Killian and Hubbard, 2005). Due to the tight conjugation between the sheath cells and the germline, completely separating them without damage was difficult. Thus, we collected sheath cells along with the segments and oocytes (Materials and Methods). Nonetheless, this also presented us an opportunity to investigate the transcriptome of the sheath cells if we were able to decipher *in silico* whether the expression of a gene originated from the germline or from the surrounding somatic tissues. As zygotes were often released in the medium once a cut was made across the vulva, and were always collected without obvious objects wrapped around, thus we reason that the zygote sample was unlikely contaminated by surrounding somatic cells. Therefore, we postulate that genes that are detected in proximal oocytes (-1 to -3) but absent in zygote samples are likely from sheath cells, and find many genes meet this criterion, such as those in clusters 7–9 (Figure 3). For instance, the expression levels of *itr-1* and *let-23* were relatively stable between -2 and -1 oocytes prior to dropping significantly in the zygotes, while that of *lin*-3 remained high and relatively unchanged between proximal oocytes and zygotes (Figure 4A). The contractile activity of sheath cells begins with major sperm protein signals to the proximal oocytes, which in turn produces and releases the LIN-3 ligand that is received by the LET-23 receptor on proximal sheath cells (Miller et al., 2001). The LET-23 receptor then triggers signaling inside the sheath cells through PLC-3, which phosphorolyzes IP3 that binds to ITR-1 receptors on the ER, causing the release of calcium (Yin et al., 2004). Moreover, sheath cell specific innexin channel encoding genes *inx*-8 and *inx*-9 (Starich et al., 2014) maintained intermediate expression levels in S1∼S4 stages, and were highly upregulated in proximal oocytes, but had negligible expression levels in the zygotes (Figure 4B). Furthermore, expression levels of sheath cell contractile activity related genes were also progressively increased along the gonadal development axis, but almost vanished in zygote samples, such as genes *pat*-10, *mup*-2, *tni*-1 and *unc*-27 coding for the troponin complex (Ono and Ono, 2004; Obinata et al., 2010) (Supplementary Figure S16A), and genes *unc*-54 and *myo*-3 coding for the myosin heavy chain (MHC) (Shelton et al., 1999; Ono and Ono, 2016) (Supplementary Figure S16B).

**FIGURE 5:**
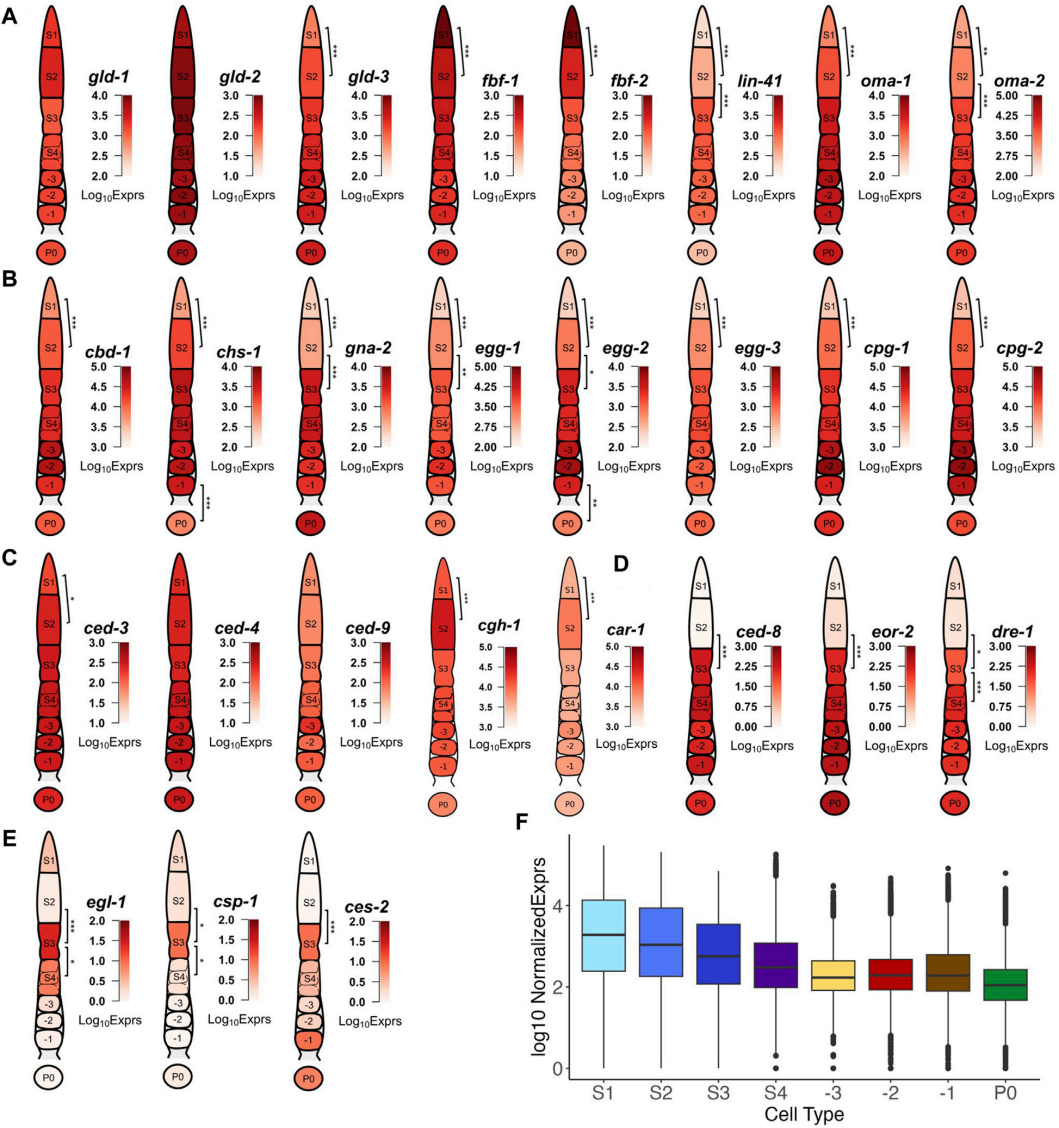
Examples of transcription of DEGs that are involved in key events of oogenesis and fertilization. **(A)** Genes encoding different elements of the eggshell. **(B)** Genes involved in mitosis-meiosis transition and meiotic maturation. **(C)** Genes involved in apoptosis with expression throughout the gonad. **(D)** Genes involved in apoptosis with elevated expression starting from the S3 stage. **(E)** Genes involved in apoptosis showing transient expression in the S3 stage. **(F)** Boxplot of transcription levels of genes coding for ribosomal subunit across each stage of gonad development and in zygotes. BH p-adj: * <0.05; ** <0.01; *** <0.0001.

Similar reasoning can be made with other genes that have evidence of somatic or germline origins. Searching the CenGEN database (Hammarlund et al., 2018) revealed genes *perm-2/4*, which encode components of the eggshell (Gonzalez et al., 2018), had the highest expression levels in sheath cells. Expression of *perm-2/4* was absent in P0 but high in -1 oocytes (Supplementary Figure S16C), while genes *egg-1/2*, which encode other known components of the eggshell that are produced in the germline (Kadandale et al., 2005), did not exhibit such significant decrease of expression in P0 (Figure 5B) (Kadandale et al., 2005). The expression of *mlc-1/3*, which are myosin light chains genes involved muscle activity (Moerman et al., 1997; Rushforth et al., 1998) through regulation of the ATPase activity of the MHCs were all high in -1 oocytes but absent in P0, while the expression of *mlc-4*, *a* non-muscle myosin light chain gene that is required for cytokinesis in zygotes, was present in P0 (Supplementary Figure S16D) (Shelton et al., 1999; Ono and Ono, 2016). Analysis of actin genes *act-1/2/3/4* (Ono and Pruyne, 2012; Ono, 2014) finds *act*-4 expression level increased in the -1 oocyte and subsequently significantly downregulated in zygotes (Supplementary Figure S16E). Considering the previous finding that *act-1/2/3* were expressed in both muscle and non-muscle cells, while *act-4* was expressed predominantly in body wall muscle (Stone and Shaw, 1993; Willis et al., 2006), our results suggest that the increased expression of *act*-4 seen in -1 oocyte samples may be primarily of sheath cell origin, and that changes in *act*-4 expression may reflect differences in sheath cells.

### Proximal oocyte expression profiles reveal potential interactions between the germline and the spermathecae

We compared the distributions of log_2_FC values for all the DEGs that exhibit significantly lower expression levels in zygotes (P0) compared to -1 oocytes. As shown in Figure 4C, a considerable number of the genes show a significant increase in expression between the -2 vs -1 comparison, as indicated by an additional small peak with higher Log_2_FC values in the distribution compared to other comparisons. These genes are largely those in clusters 7 and 8 (Figure 3). This is interesting, as early studies indicate transcriptional inactivity or an overall presence of transcriptional silence as oocytes move to the proximal end (Starck, 1977; Walker et al., 2007). Though this was the case between the -3 and -2 oocytes, it was clear that there were significant differences in transcripts detected between the -2 and -1 oocytes (Figure 2B). This expression pattern appeared different from expression patterns of genes of gonadal sheath origin that we described earlier, where the expression levels stay relatively stable in the proximal oocytes. We took notice of two Uterine Lumen-Expressed (ule) genes *ule*-3 and *ule*-5 (Figure 4D), which exhibited sudden increases in transcription from 10-fold to 100-fold between the -2 and -1 oocyte transition. It has been reported that *ule*-3/5 might play a role in driving the ageing of the reproductive system, though the origin of their expression is not clearly discernible (Zimmerman et al., 2015). A more recent study utilizing fluorescent *in situ* hybridization (FISH) to track the origins of these transcripts suggests a mechanism by which the transcripts are produced in spermathecae and carried over into the proximal oocytes (Trimmer et al., 2023). Using the expression of *ule-*3/5 as a reference, we discerned a set of 25 genes displaying the similar expression pattern (Supplementary Table S7) by invoking a stringent criterion: Log_2_FC < −7 in the -1 vs. P0 comparison; and Log_2_FC > 4 in the -2 vs -1 comparison; and average Median Normalized Expression in -1 oocytes >1,000. This criterion also allows us to potentially filter out genes with increased expression due to differences between proximal sheath cells, as those expression changes may not be as drastic. Of these 25 genes, 17 have ISH images the NEXTDB database (Kohara, 2001), of which 13 (including ule-3) exhibited clear localization of transcripts in the spermathecae region (Figure 4E; Supplementary Figure S7). These results suggest possible interactions between transcriptionally silent oocytes and their somatic neighbors, where transcripts in the surrounding spermathecae might be transferred into the proximal oocytes and rapidly depredated in the zygotes.

### DEGs mark transcriptional timing of the key events of oogenesis and fertilization

One of the early key events in the oogenesis process is the control of mitosis and meiosis. Thus, it is interesting to look into the transcription patterns of GLD-1/2/3, which promote germline differentiation (Eckmann et al., 2004), and the RNA binding proteins (RBP) FBF-1/2, which maintain mitosis in the distal germline via inhibition of GLD activities (Crittenden et al., 2002). We found that *gld*-1/2 maintained high expression along the entire gonad developmental axis, while *gld*-3 exhibited increased expression between S1 and S2, and the levels were maintained thereafter (Figure 5A). Expression of *fbf-1/2* showed was high in the S1 stage, but subsequently decreased in the S2 and S3 stages and beyond (Figure 5A). In addition to maintaining mitosis in the distal gonad via repression of transcripts of meiosis promoting gene *gld-1 and gld-3* (Crittenden et al., 2002; Hansen et al., 2004), FBF-1/2 regulate one another and function antagonistically for the transition between mitosis to meiosis, with FBF-2 promoting meiotic entry (Wang et al., 2020; Albarqi and Ryder, 2023). These results suggest transcript level regulation of *fbf-1/2* may contribute to the translational de-repression of meiosis promoting factors. Furthermore, we observed transcriptional changes of key genes involved in the maintenance of germ cells in meiotic prophase I such as OMA-1/2 and LIN-41 (94). Specifically, the expression of *oma*-1/2 and lin-41 gradually increased throughout the early stages (S1 and S2) of oogenesis followed by high elevations in the S3 stage, which were maintained even after fertilization, apart from *lin*-41, whose expression dropped after fertilization (Figure 5A). It has been suggested that LIN-41 could prolong prophase I and inhibit meiotic maturation after fertilization by a translational level regulatory mechanism (Spike et al., 2014; Tsukamoto et al., 2017), thus diminishment of the *lin-41* transcripts in zygotes suggests that transcriptional degradation might also play a role in the exit of the oocyte from metaphase I upon fertilization.

We also found that many genes coding for eggshell components were upregulated in distal segments of the gonad, far before the complete formation of the eggshell that happened around the early-stage embryo (Stein, 2018). Genes coding for components of the vitelline layer (*cbd*-1) (Gonzalez et al., 2018), the chitin layer (*chs*-1, *gna*-2, *egg*-1/2/3) (Kadandale et al., 2005; Zhang et al., 2005; Johnston et al., 2006; Maruyama et al., 2007; Johnston and Dennis, 2012) and the proteoglycan layer (*cpg*-1/2) (Olson et al., 2006) all exhibit increased expression in early stages of the germline until after fertilization (Figure 5B). ISH images of *egg-1 and cpg-1/2* transcripts in NEXTDB are in good agreement with our results (Supplementary Figure S13). These results suggest that transcription of these eggshell genes occur mostly during the mitosis to meiosis transition and the pachytene, while translation and degradation of these transcripts might occur as a response to fertilization signaling.

Moving along the germline, another key event of oogenesis happens in the diplotene loop (S3) where germ cells undergo apoptosis (Gartner et al., 2008). Interestingly, we find that genes regulating apoptosis form three distinct patterns of expression. The expression of genes encoding core apoptosis machinery such as apoptosis initiators CED-4/3 (103) and apoptosis inhibitor CED-9 (Hengartner et al., 1992) were relatively stable in the distal gonad (with only ced-3 exhibiting increase of expression between S1 and S2) in (Figure 5C). The high expression levels of ced-3/4/9were largely maintained thereafter (Figure 5C). Two other genes *cgh-1* and *car-1*, RNAi depletion of which have been found to contribute to increased physiological apoptosis in the germline (Navarro et al., 2001; Boag et al., 2005), show significant increased expression in S2 and slight decrease of expression in S3. Our results suggest that levels of *car-1* and *cgh-1* may be partially transcriptionally downregulated to induce apoptosis in a portion of the germ cells.

Expression of *ced*-8, which encodes a transmembrane protein likely involved in apoptosis dynamics and functions as a cell death effector downstream of the CED-3 Caspase (Stanfield and Horvitz, 2000; Chen et al., 2013), follows a different pattern with significant upregulation in the S3 stage, and maintaining high expression until fertilization (Figure 5D). The sudden increase in *ced*-8 transcription in the S3 stage suggests that CED-8 might play an important role in promoting cell killing via phagocytosis during germline apoptosis (Chen et al., 2013). Other genes such as *eor*-2 and *dre*-1 showed expression patterns like that of *ced*-8, with elevated expression starting from the pachytene (S3) loop onwards through fertilization (Figure 5D). DRE-1 has been found to interact directly with CED-9 in regulating apoptosis (Chiorazzi et al., 2013). Early studies have found EOR-2, along with EOR-1 to induce apoptosis in neuronal cells (Hoeppner et al., 2004). However, we only observed upregulation of *eor-2* (Figure 5D) but not of *eor-1* in the germline, suggesting a possibly different mechanism of EOR-2 induced apoptosis in the germline than in neuronal cells.

The third group of apoptosis related genes follow a different expression pattern that can be characterized by the expression profile of *egl-1*, which encodes a direct downstream target of CED-4 and an inhibitor of CED-9, thus playing a critical role in DNA damage induced germline apoptosis (Huang et al., 2013). *Egl-1* exhibited a transient increase in transcription in the pachytene loop (S3) that did not go beyond the S4 stage (Figure 5E). Other apoptosis related genes such as *csp*-1 and *ces*-2 displayed expression patterns like that of *egl*-1 (Figure 5E). Consistently, an earlier study found that *csp-1* was expressed in late stage pachytene of the germline using FISH imaging (Denning et al., 2013). *Ces-2* has been implicated in the apoptosis of neuronal cells in *C. elegans*, though a previous study suggested that *ces*-2 was not essential for germline apoptosis (Metzstein et al., 1996). However, the sudden upregulation of *ces-2* transcription in S3 strongly suggests a role of *ces-2* in apoptosis of the germline. Furthermore, since all 3 genes belong to cluster 14 (Figure 3), it is likely cluster 14 contains other genes that are related to apoptosis as well.

As shown in Figure 5F, genes encoding ribosome subunits and other translation-related proteins generally exhibited downtrends in transcription as oocytes matured and prepared for fertilization, consistent with a previous observation (Diag et al., 2018). Though downregulation occurs early on in the pachytene, the most significant downregulation occurs between S4 and -3 and includes many ribosomal protein subunits, such as *rla-0/1*, *rpl-1/2/3/4/5/7/9/10/13/14/15/16/17* and *rps-0/1/2/3/4/5/7/8/9/10/11/12/13/14/15* (Supplementary Table S3) (Nakao et al., 2004). Moreover, the reduced expression of ribosomal protein genes from the S4 stage and beyond suggests that the transcription of translational machinery required for oocyte maturation might have been completed before the diakinesis stage.

### Differential alternative polyadenylation activity resumes post-fertilization

**FIGURE 6:**
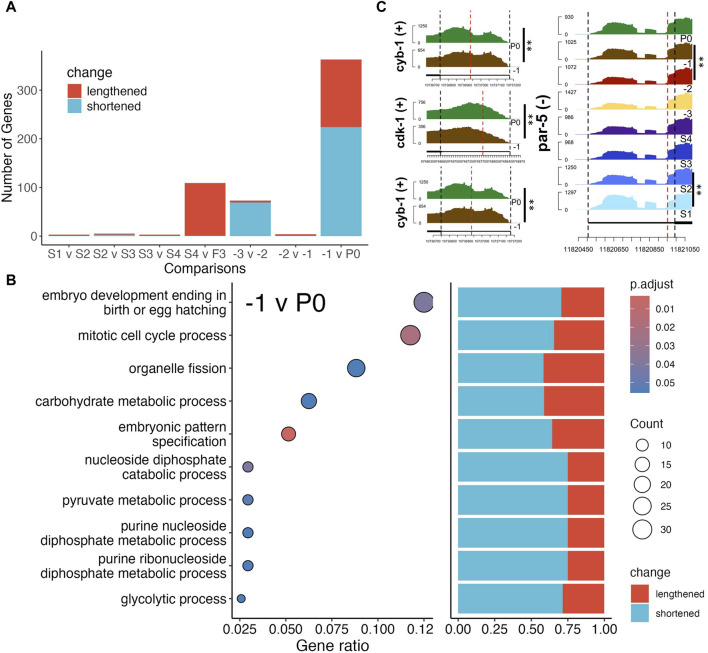
Differential alternative polyadenylation (DAP) analysis of genes between neighboring stages. **(A)** Number of DAP genes between each neighboring stage, colors indicate lengthening (purple) or shortening (yellow) of 3′UTR lengths. **(B)** GO term enrichment of significant DAP gene between F1 and P0 (left panel), and bar plot of percentage of significantly lengthened or shortened genes in each enriched gene set, GeneRatio is the proportion of differentially polyadenylated genes that belong to a known gene set. **(C)** Coverage by RNA-seq reads of 3′UTRs of genes *cyb-1/2*.*2*, *cdk-1* and *par-5*, red lines mark the estimated proximal polyadenylation site. BH p-adj: * <0.05; ** <0.01.

Though many studies focused on regulation of translation through the 3′UTRs of transcripts by RNA binding proteins (RBPs), few have elucidated changes of the 3′UTRs themselves (Merritt et al., 2008; Mangone et al., 2010; Diag et al., 2018; Steber et al., 2019). Thus, we analyzed differential alternative polyadenylation (DAP) usage through the DaPars2 software (Feng et al., 2018; Li et al., 2021), which estimates changes in proportion of distal (lengthened 3′UTR) and proximal (shortened 3′UTR) polyadenylation sites used under two different conditions. We found very few significant changes in distal versus proximal sites usage between neighboring stages, apart from the S4 vs. -3, -3 vs. -2 and -1 vs. P0 comparisons (Figure 6A). GO term enrichment analysis found that only the -1 vs. P0 comparison resulted in significant enrichment of genes with DAP for mitotic cell cycle related processes, mostly with shortened 3′UTRs (Figure 6B). For instance, we found that *cyb*-1/2.2 exhibited shortened 3′UTRs while *cdk*-1 exhibited lengthened 3′UTR (Figure 6C). CYB-1/2.2 along with CDK-1 regulate M phase entry of cell cycle in *C. elegans* (Rabilotta et al., 2015). Though most DAP genes between -1 oocytes and P0 exhibit shortened 3′UTR (Figure 6B), it is not straightforward how usage of distal vs. proximal sites regulates protein production. Furthermore, despite very few significant DAP genes in the early stages of oogenesis, we found the *par*-5 gene to exhibit DAP in both the S1 vs. S2 and -2 vs. -1 comparisons (Figure 6C). In fact, 3′UTR length of the *par*-5 transcript gradually decreased until the -3 stage before increasing again afterward (Figure 6C). Previous studies found that PAR-5 regulates asymmetric cell division and alternative 3′UTR isoforms of *par-*5 confers different levels of the PAR-5 protein (Mikl et al., 2014). Interestingly, most genes with DAP between S4 and -3 oocytes exhibited an increase in 3′UTR length, while most genes with DAP between -3 and 2 oocytes exhibited decreased in 3′UTR length (Figure 6A). However, it is unclear whether this is because -3 oocytes are fully cellularized and maintain a stable transcriptome or other factors.

### Differential splicing play roles in germline development

**FIGURE 7:**
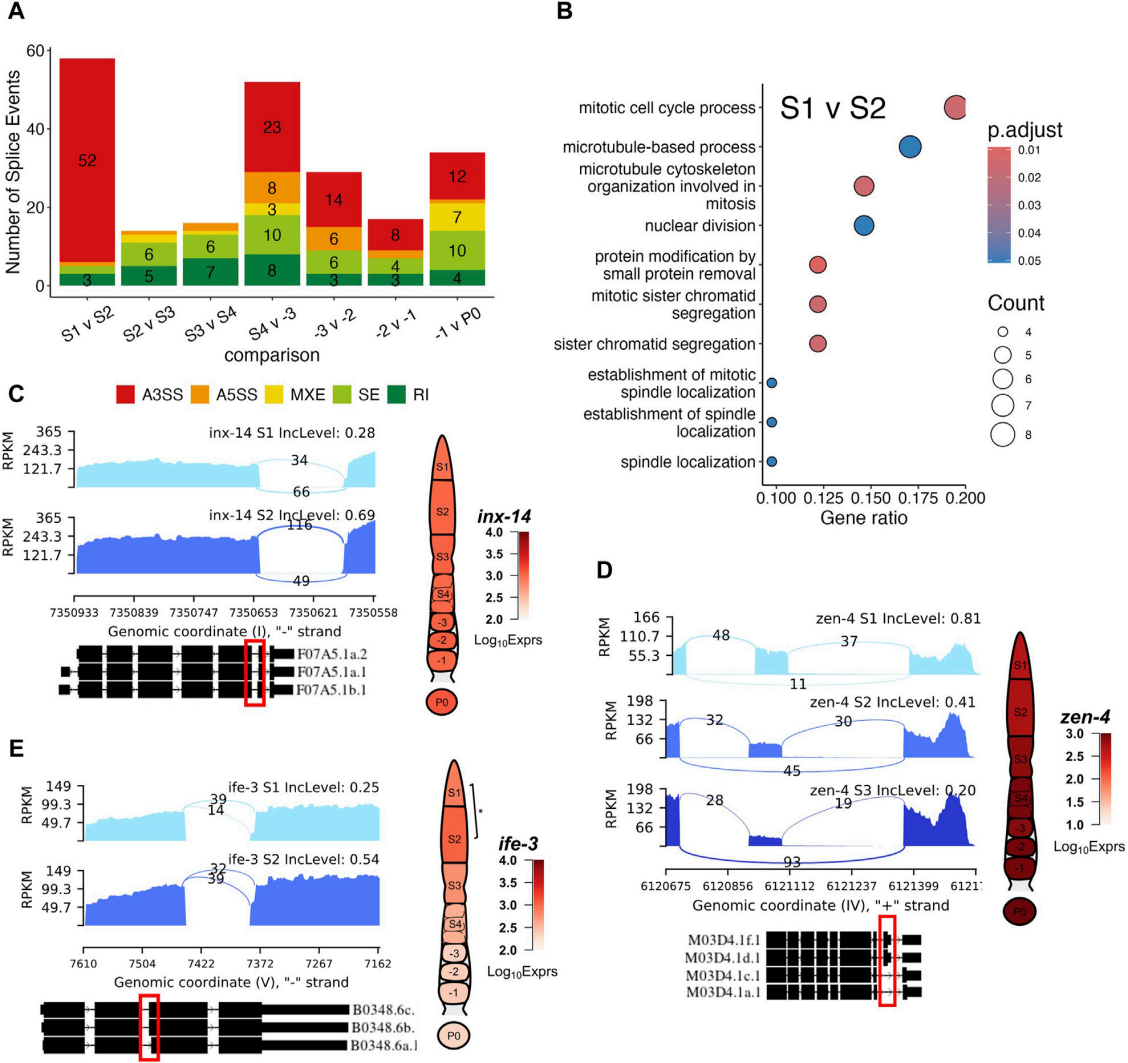
Examples of differential splicing usage of genes during germline development. **(A)** Box plot of numbers of five splicing types (A3SS, A5SS, MXE, SE and RI, see main text for definitions) detected between each pair of neighboring stages of germline development. **(B)** Enriched GO terms of genes with differential splicing events between the S1 and S2 stages, GeneRatio is the proportion of differentially spliced genes that belong to a known gene set. **(C)** Differential splicing events of gene *inx*-14 between the S1 and S2 stages. **(D)** Differential splicing events of gene *zen*-4 between the S1 and S2 as well as S2 and S3 stages. **(E)** Differential splicing event of gene *ife*-3 between the S1 and S2 stages. Exact positions of splicing events are shown in the red box. BH p-adj: * <0.05; ** <0.01; *** <0.0001.

We further performed differential splicing analysis using rMATs (Shen et al., 2014) to look for differential transcription of alternatively spliced isoforms of genes between neighboring stages along the oocyte developmental axis. Since rMATs could not account for batch effects, we performed the analysis with samples from Batch A (Figure 2A). We identified varying numbers of genes exhibiting significant splicing signals defined by rMATs, i.e., alternative 3′ splice site (A3SS), alternative 5′ splice site (A5SS), retained intron (RI), mixed exon usage (MXE), skipped exon usage (SE), between neighboring stages. Most notably, the S1 vs. S2 and the S4 vs. -3 oocyte comparisons yielded the most differential splicing usage with 58 genes and 54 genes exhibiting differential splicing, respectively (Figure 7A). Genes with differential splicing usage between the S1 vs. S2 comparison are enriched for GO terms related to mitosis (Figure 7B), which is expected, given the fact that S1 contains the TZ regions (Figure 1A). However, other neighboring stages comparisons yielded no significantly enriched GO BP terms. A few interesting examples are detailed as follows.

Gene *inx-*14 was differentially spliced during the S1 to S2 transition (Figure 7C). Specifically, *inx-*14 was preferentially utilized for its longer sixth exon in the S2 stage compared to the S1 stage, resulting in increased proportions of its F07A5.1b isoform (Figure 7C). It has been documented that germline innexins INX-14/INX-21 and somatic innexins INX-8/9 forms gap channels that facilitate the communication between the somatic gonad and the distal germline to promote germline proliferation (Starich et al., 2014). UniProt designates the F07A5.1b isoform as the canonical isoform, differing from the alternative F07A5.1a isoform by 2 amino acids in the 406-407 positions. Our results suggest a possible mechanism by which INX-14 functions are regulated. (Starich et al., 2014).

Another notable event was a gradual increase in preference of *zen-4* skipping its eighth exon in the S1 to S3 transition (Figure 7D). Specifically, *zen*-4 is predominantly spliced in mutlple isoforms, including M03D4.1a.1, M03D4.1c.1, M03D4.1d.1 and M03D4.1f.1 (Figure 7D). This is due to the lack of read coverage for the regions that are spanned by the other isoforms (Figure 7D). The exon skipping event is indicative of decreased preference for the M03D4.1d.1 and M03D4.1f.1 isoforms, which contain the skipped exon in the other isoforms (Figure 7D). ZEN-4 along with CYK-4 forms the central spindlin complex, a conserved component of intercellular bridges that function in cellularization of oocytes during cytokinesis (White and Glotzer, 2012; Zhou et al., 2013; Lee et al., 2018). Though a recent study suggested that ZEN-4 was not essential in the germline for the closure of the intercellular bridge (Lee et al., 2018), our results suggest that as the oocyte moves along the rachis into late pachytene stage, alternative isoforms of *zen*-4 may play a role in the cellularization of maturing oocytes.

In addition, we found that *ife*-3 switched isoforms during the S1 to S2 transition (Figure 7E). The *ife*-3 gene encodes one of the *C. elegans* homologs for human translation initiation factors (eIFs) that play critical role in regulating mRNA content along with microRNA and RBPs binding proteins (Huggins et al., 2020). More specifically, *ife*-3 functions as a repressor of *fem*-3 expression to promote production of oocytes in the germline (Mangio et al., 2015; Huggins et al., 2020). Here, we showed a switch in *ife*-3 splicing preference for the B0348.6b and B0348.6c isoforms over the shorter B0348.6a isoform (Figure 7E). Along with a slight increase in *ife*-3 expression, these results hint at a possible mechanism of *ife*-3 regulation in the pachytene stage of oogenesis. Interestingly, *ife*-3 expression reduced significantly in proximal oocytes, where transcription became increasingly silent, thus obviating the need for mRNA regulation (Figure 7E).

Other genes worth pointing out include *tos-1* coding for a reporter of differential splicing (Ma et al., 2011), and *lev*-11 coding for tropomyosin (Watabe et al., 2018). Transcripts of *tos*-1 exhibit loss of preference for the usage of its third exon from -1 to zygote transition (Supplementary Figure S18A), which is further corroborated by the decreased coverage of its longer isoform in S4 (Supplementary Figure S18A). As we described above, *lev-11* belongs to cluster 9 and its expression exhibits similar putative somatic characteristics. Thus, differential splicing of *lev*-11 transcripts (Supplementary Figure S18B) might be occurring in the sheath cells wrapped around the oocytes. It has been shown that different isoforms of *lev*-11 exhibit different characteristics in terms of muscle assembly and function (Watabe et al., 2018). Since -2 oocytes are roughly covered by Sh4 and -1 oocytes by Sh5, it is likely that an alternative isoform switch of *lev-*11 contributes to the different functions of these two sheath cell types.

## Discussion

With a spatial layout of cells that simultaneously mirrors the timeline of oogenesis, the *C. elegans* gonad can serve as a powerful model for uncovering mechanisms of oogenesis. However, the tiny size of the gonad also presents challenges for in-depth studies of the intricacies of this process. With the recent development of single cell methods, we utilize scRNA-seq techniques to decipher the transcriptomic landscape of different stages of oocyte formation as well as fertilization, though the data from the S1-S4 segments are not of single cell resolution as they contain multiple nuclei due to their syncytial structure. Our transcriptomic dataset of the *C. elegans* gonad presents a good resource for research into the transcriptional landscape of oogenesis of animals. Our results not only are able to recall most of the oogenic genes designated by earlier research that utilized micro-arrays and bulk-RNAseq (Reinke et al., 2004; Ortiz et al., 2014), but also are highly correlated with the earlier data, through careful alignment of samples, especially with those generated using single cell based techniques (Diag et al., 2018; Tzur et al., 2018).

Although care has been taken, the dissection of the tiny gonad presents a delicate problem, and it is difficult to fully avoid contamination by surrounding tissues, particularly, sperm and intestines. Based on known genes that are specifically transcribed in surrounding tissues, we were able to filter out these genes, and mitigating their impacts on our analyses. Though our dataset presents discernible batch effects, we either incorporated them into our analysis models or forfeited the smaller batch of samples when necessary. The number of biological repeats for each stage as well as the sequencing depth for each sample means the results are robust to the discarding of few samples. The expression profiles of the samples in both batches show a trajectory pattern in the UMAP display, which is consistent with the developmental axis of the gonad, indicative of our successful capture of the transcriptomes underlying the oogenesis program. Moreover, clustering analysis of DEGs identified between adjacent stages reveals 20 distinct patterns of dynamic changes in their expression along the gonad developmental axis. Expression patterns of the majority of randomly selected DEGs in the clusters are in excellent agreement with ISH images of the transcripts of corresponding genes in the NEXTDB database (Shin-i and Kohara, 1999), indicating that our results are of high accuracy.

We note that distal stages (S1–S3) inevitably contain transcripts originating from Sh1 and Sh2 sheath cells, due to the unenclosed and miniscule nature of the germline along the rachis. Thus, we focus on elucidating the transcriptomic changes of known germline associated genes to minimize false positive findings. On the other hand, the difficulty to remove to sheath cells wrapped around proximal stage oocytes prompted us to investigate patterns of expression that may arise from known sheath cell specific genes and investigate interactions between proximal oocytes and surround somatic tissues. We find that a great portion of genes that are drastically downregulated in zygotes relative to the -1 oocyte are of somatic origin, including many known markers of muscle cells and sheath cells. This allowed us to infer a large portion of genes as somatic in nature, especially those in the proximal oocytes. From their expression patterns throughout the gonad, these genes can be divided into roughly two groups. The first group consist of genes that have relatively stable transcription in the proximal oocytes before complete disappearance in zygotes, and the second group consist of genes that are drastically upregulated in only the -1 oocyte. The second group include genes whose transcripts have recently been found to be produced in the spermathecae but transported into -1 oocytes (Trimmer et al., 2023). We thus provide a list of genes that might undergo this process. Though the exact function and underlying mechanism for this phenomenon remain to be elucidated, a few genes exhibiting this pattern have been shown to affect the ageing of *C. elegans* (Zimmerman et al., 2015).

We confirm previous findings (Lee et al., 2012) at the transcriptomic level that the growth of oocytes presents as a process in which ribosomal biogenesis and cellular activity gradually decreases. Moreover, we observed at the transcriptional level known dynamics of core regulators of the mitosis to meiosis switch and meiosis maturation. In addition, we find that many genes involved in the eggshell formation initiate transcription as early as in the S1 stage, and their transcripts are accumulated until post fertilization. The *C. elegans* germline also presents a remarkable model for studying germ cell apoptosis (Gumienny et al., 1999). Our results not only capture distinct upregulation of apoptosis related genes in the pachytene loop (S2 stage), but also discover novel candidate genes for future studies of germ line apoptosis. Furthermore, our gene clustering and DEG results also reveal three distinct sets of apoptotic related genes, characterized by the expression pattern of the *ced*-3/4/9 (Figure 5C), *ced*-8 (Figure 5D) and *egl*-1 (Figure 5E) genes, respectively. These different modes of transcription suggest that cross-talks occur between different genes at the transcriptional and post-transcriptional levels to induce apoptosis.

The previous report that RBPs and the 3′UTRs are key players in a complex regulatory mechanism (Merritt et al., 2008; Mangone et al., 2010; Diag et al., 2018) in the *C. elegans* germline prompted us to investigate whether significant changes in polyadenylation site usage occurred during oogenesis and fertilization. Though we are not able to find significantly enriched pathways regulated via changes in polyadenylation during oogenesis, we do find enrichment for cell cycle processes due to changes in polyadenylation site usage during fertilization. Our results suggest that polyadenylation sites of transcripts remain relatively stable during oogenesis, and active regulation of alternative polyadenylation likely resumes in the zygote.

Finally, we find that alternative splicing events are present throughout the gonadal segments. We reveal significant changes in the usage of isoforms of hemi-channel gene *inx*-14. It is highly likely that products of different isoforms of *inx*-14 are associated with germline hemichannels INX-21/22 or somatic hemichannels INX-8/9 to facilitate communication between the somatic gonad and germline. We also find differential splicing usage of genes in the germline. For instance, we observe differential splicing of *zen-*4 throughout the pachytene region. Though previous studies preclude the involvement of ZEN-4 in oocyte cellularization in the germline syncytium (Lee et al., 2018), ZEN-4 isoforms may play roles in oocyte growth in late pachytene. Future studies are needed to elucidate the roles of isoform usages in *C. elegans* oogenesis and the underlying mechanisms.

Taken together, our results paint a complex transcriptional landscape of the germline development, oogenesis and fertilization processes in *C. elegans* in finer detail than previous studies. Though contaminations of sperm and somatic tissues present challenges, we were able to largely filter them out *in silico*, and meanwhile discern putative somatic elements. We not only confirm previous findings, but also reveal many novel transcriptional events along the temporospatial axis of the *C. elegans* germline and in the zygote. Our study presents a wealth of resources and gene candidates for future experimental investigation to reveal the underlying mechanisms of the oogenesis program, although much work remains to be done. Particularly, improving dissection techniques to sufficiently remove somatic contaminations may allow better delineation of the oogenesis program as well as provide transcriptomic profiles of the somatic gonad. Along the same line of thought, single nucleus transcriptomics may provide fine grain profiling of every oocyte along the gonad, albeit *in silico* separation and re-alignment of samples to a developmental trajectory may be required. Finally, application of a multi-omics approach on individual cell/stages of the gonad may paint a more comprehensive picture of the regulatory relationships that drive oogenesis.

## Lead contact

Further information and requests for resources and reagents should be directed to and will be fulfilled by the lead contact, Zhengchang Su (zcsu@charlotte.edu).

## Data Availability

The datasets presented in this study can be found in online repositories. The names of the repository/repositories and accession number(s) can be found below: https://www.ncbi.nlm.nih.gov/geo/, GSE261784.
