# Supplementary material for: Transcriptomic analysis of the spatiotemporal axis of oogenesis and fertilization in C. elegans
Source: Front Cell Dev Biol. 2024 Aug 19;12:1436975. doi: 10.3389/fcell.2024.1436975 (PMC11366716; doi:10.3389/fcell.2024.1436975)
Supplement: Supplementary file 1 [file DataSheet1.pdf]

Supplementary Materials

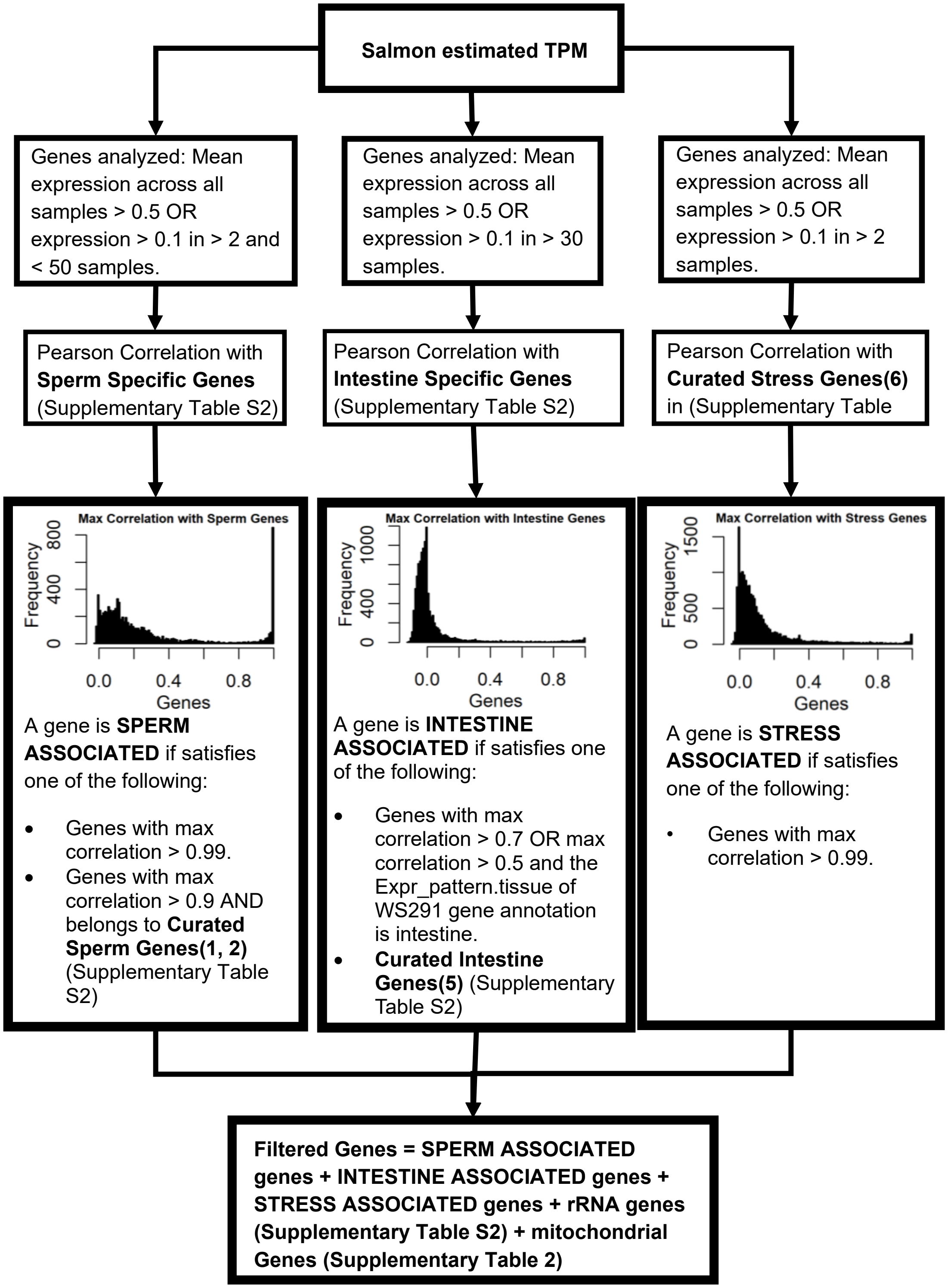

Supplementary Figure S1 Gene filtering pipeline for mitochondrial, rRNA, intestine, sperm and stress associated genes. WS291 gene annotations are obtained via the Wormbase SimpleMine tool(3, 4).

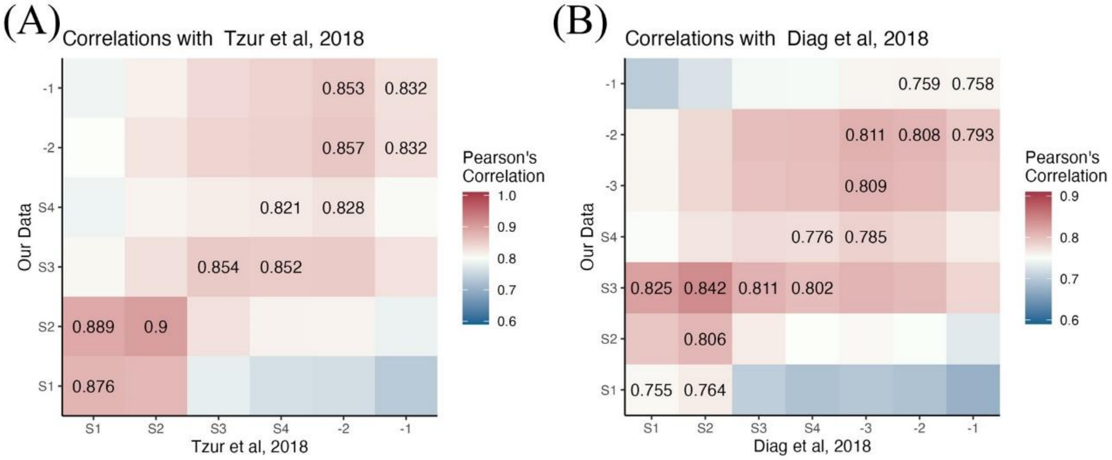

Supplementary Figure S2 A, B. Heatmap of Pearson correlation coefficient of our detected expressed genes in the segments with those of Tzur et al. (A) and of Diag et al. (B).

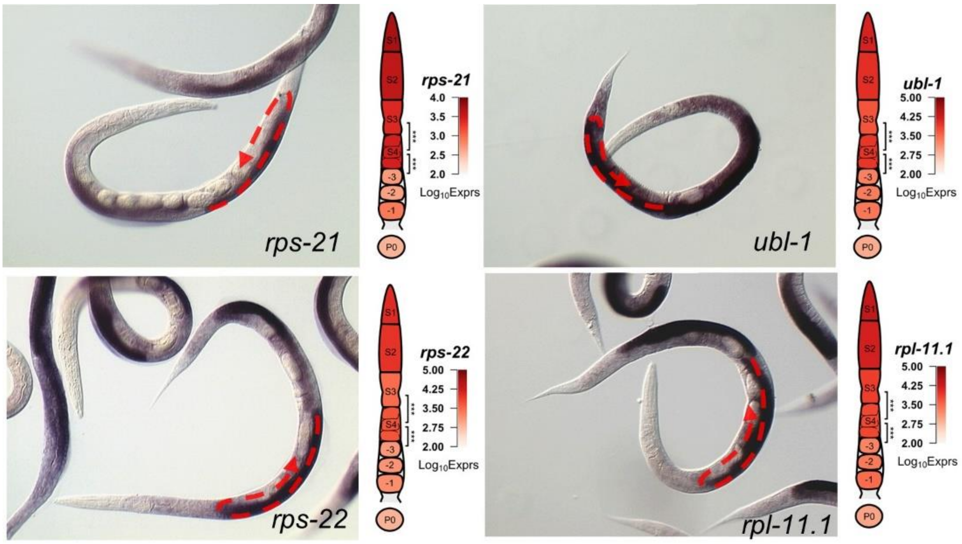

Supplementary Figure S3. ISH images taken from NEXTDB(7) for *genes rps-21, rps-22, rpl-11.1* and *ubl-1* in cluster 2, dashed arrow shows oogenesis path in the gonad. BH p-adj: * < 0.05; ** < 0.01; *** < 0.0001

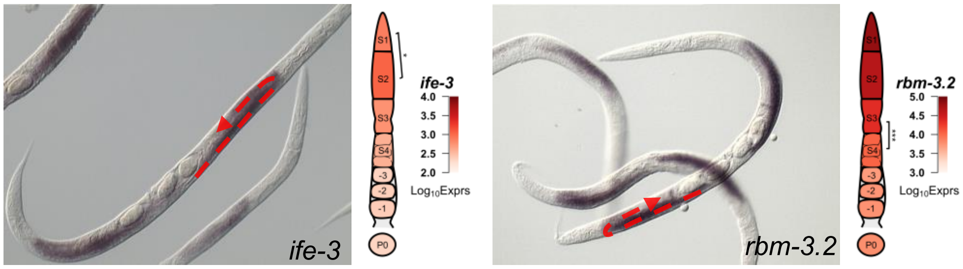

Supplementary Figure S3. ISH images taken from NEXTDB(7) for genes *ife-3* and *rbm-3.2* in cluster 3, dashed arrow shows oogenesis path in the gonad. BH p-adj: * < 0.05; ** < 0.01; *** < 0.0001

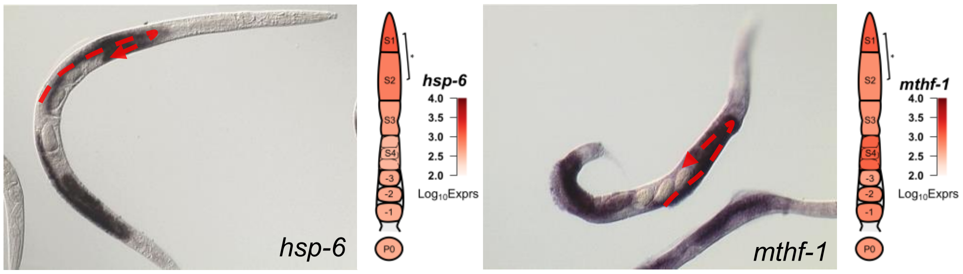

Supplementary Figure S4. ISH images taken from NEXTDB(7) for genes *hsp-6* and *mthf-1* in cluster 4, dashed arrow shows oogenesis path in the gonad. BH p-adj: * < 0.05; ** < 0.01; *** < 0.0001

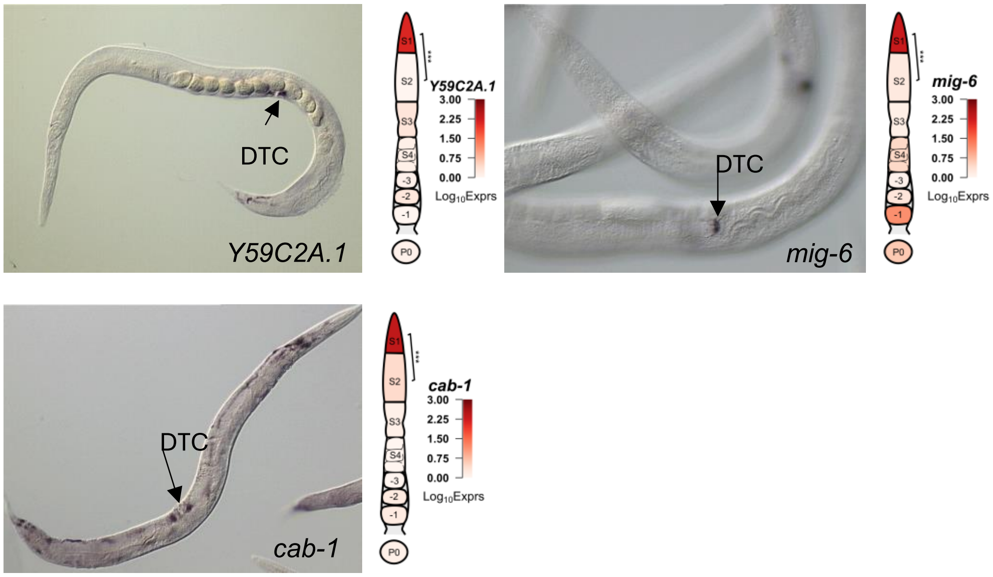

Supplementary Figure S5. ISH images taken from NEXTDB(7) for genes *Y59C2A.1, mig-6* and *cab-1* in cluster 5, arrow points to DTC location. BH p-adj: * < 0.05; ** < 0.01; *** < 0.0001

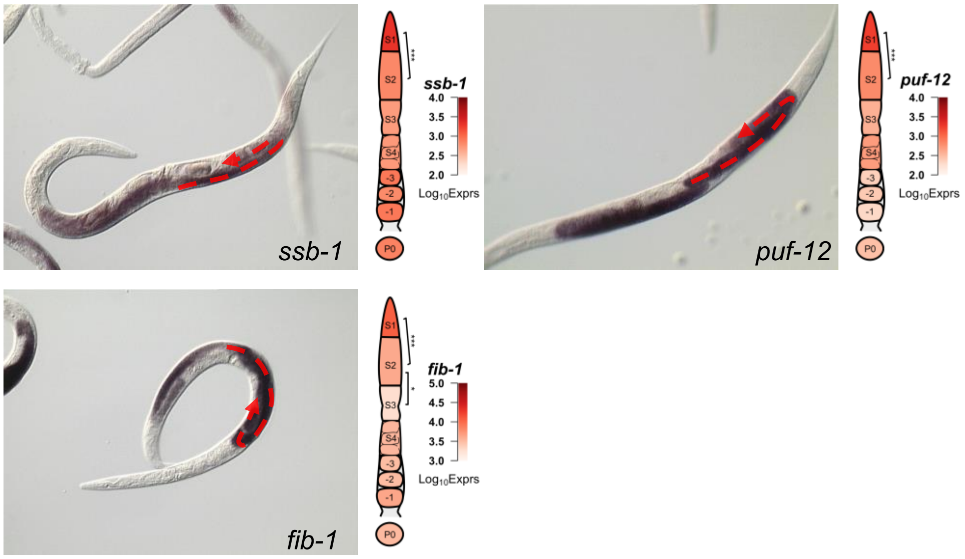

Supplementary Figure S6. ISH images taken from NEXTDB(7) for *genes ssb-1, puf-12* and *fib-1* in cluster 6, red dashed arrow shows oogenesis path in the gonad. BH p-adj: * < 0.05; ** < 0.01; *** < 0.0001

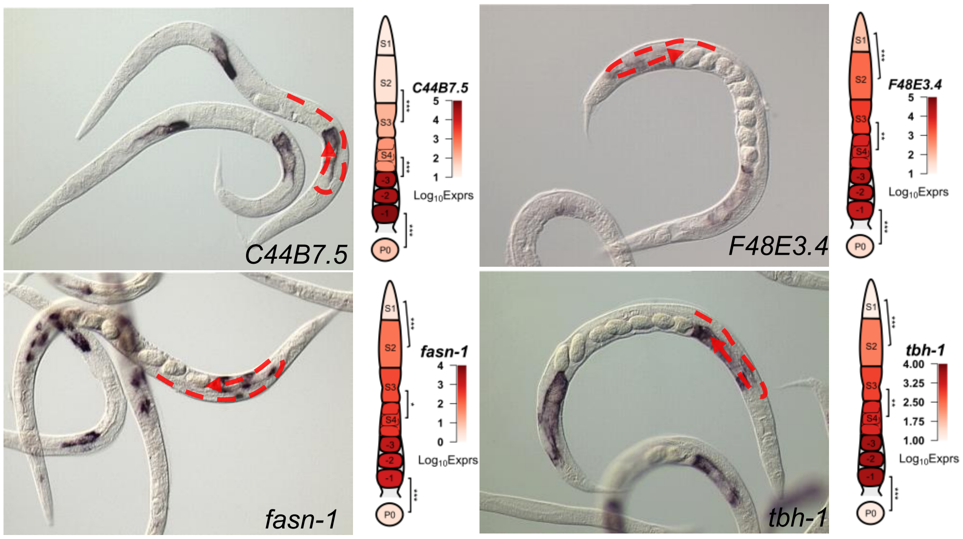

Supplementary Figure S7. ISH images taken from NEXTDB(7) for genes *C44B7.5, F48E3.4, fasn-1* and *tbh-1* in cluster 9, red dashed arrow shows oogenesis path in the gonad. BH p-adj: * < 0.05; ** < 0.01; *** < 0.0001

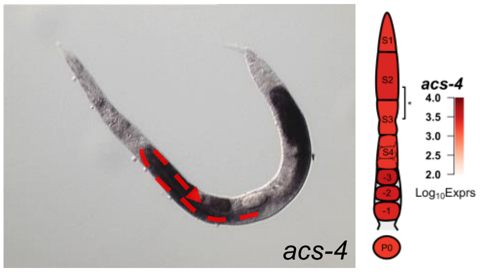

Supplementary Figure S8. ISH images taken from NEXTDB(7) for *acs-4* in cluster 10. Dashed red arrow shows oogenesis path in the gonad. BH p-adj: * < 0.05; ** < 0.01; *** < 0.0001

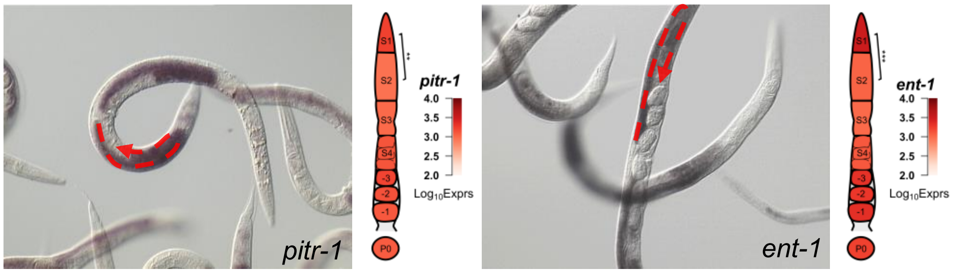

Supplementary Figure S9. ISH images taken from NEXTDB(7) for genes *pitr-1* and *ent-1* in cluster 11. Dashed red arrow shows oogenesis path in the gonad. BH p-adj: * < 0.05; ** < 0.01; *** < 0.0001

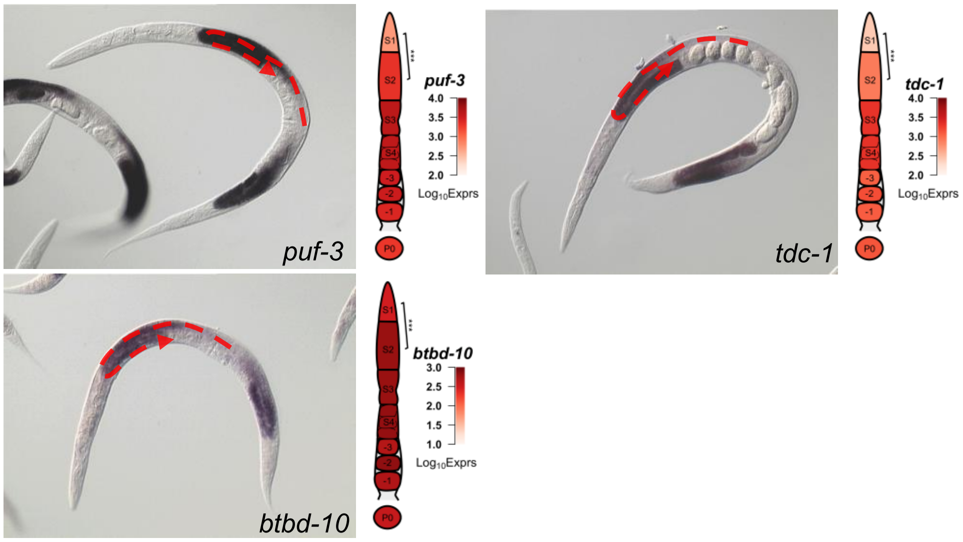

Supplementary Figure S10. ISH images taken from NEXTDB(7) for genes *puf-3, tdc-1* and *btbd-10* in cluster 15. Dashed red arrow shows oogenesis path in the gonad. BH p-adj: * < 0.05; ** < 0.01; *** < 0.0001

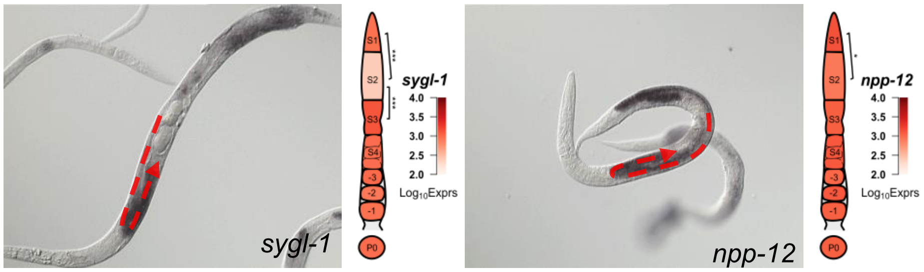

Supplementary Figure S11. ISH images taken from NEXTDB(7) for *sygl-1* and *npp-12* in cluster 16, red dashed arrow shows oogenesis path in the gonad. BH p-adj: * < 0.05; ** < 0.01; *** < 0.0001

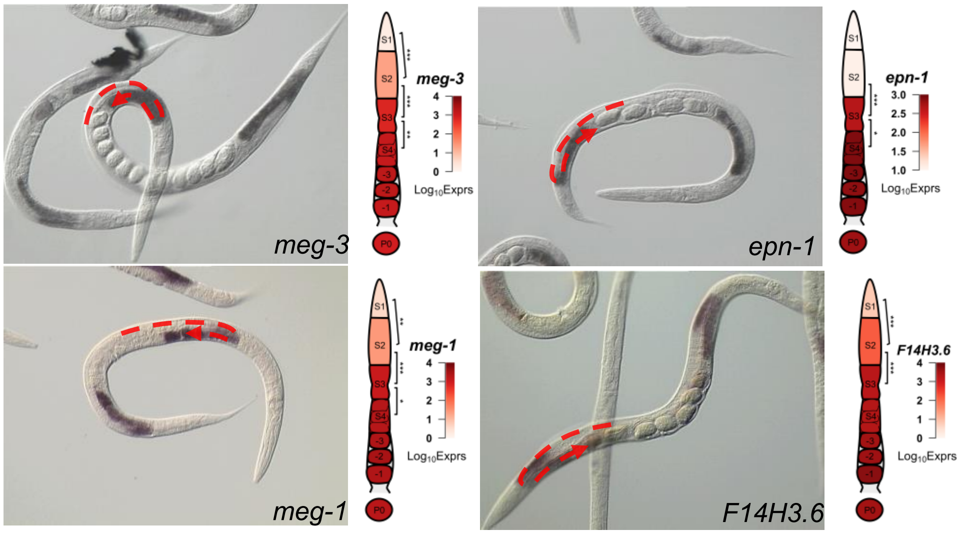

Supplementary Figure S12. ISH images taken from NEXTDB(7) for genes *meg-3, epn-1, meg-1* and *F14H3.6* in cluster 17, red dashed arrow shows oogenesis path in the gonad. BH p-adj: * < 0.05; ** < 0.01; *** < 0.0001

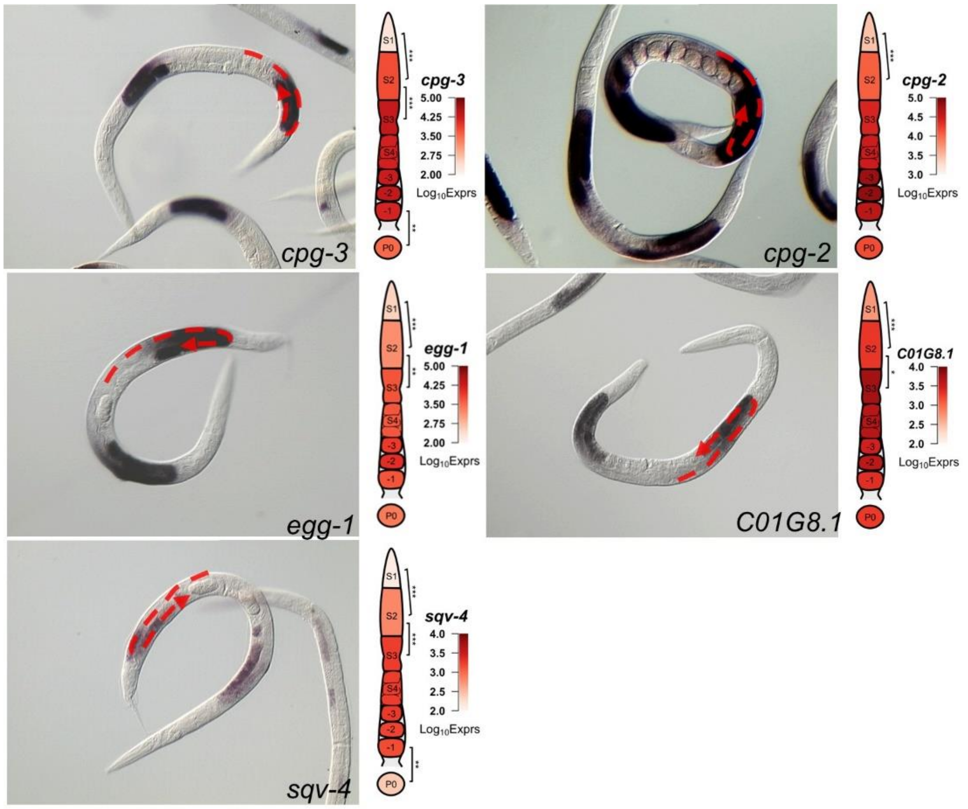

Supplementary Figure S13. ISH images taken from NEXTDB(7) for genes *cpg-3, cpg-2, egg-1, C01G8.1* and *sqv-4* in cluster 18, red dashed arrow shows oogenesis path in the gonad. BH p-adj: * < 0.05; ** < 0.01; *** < 0.0001

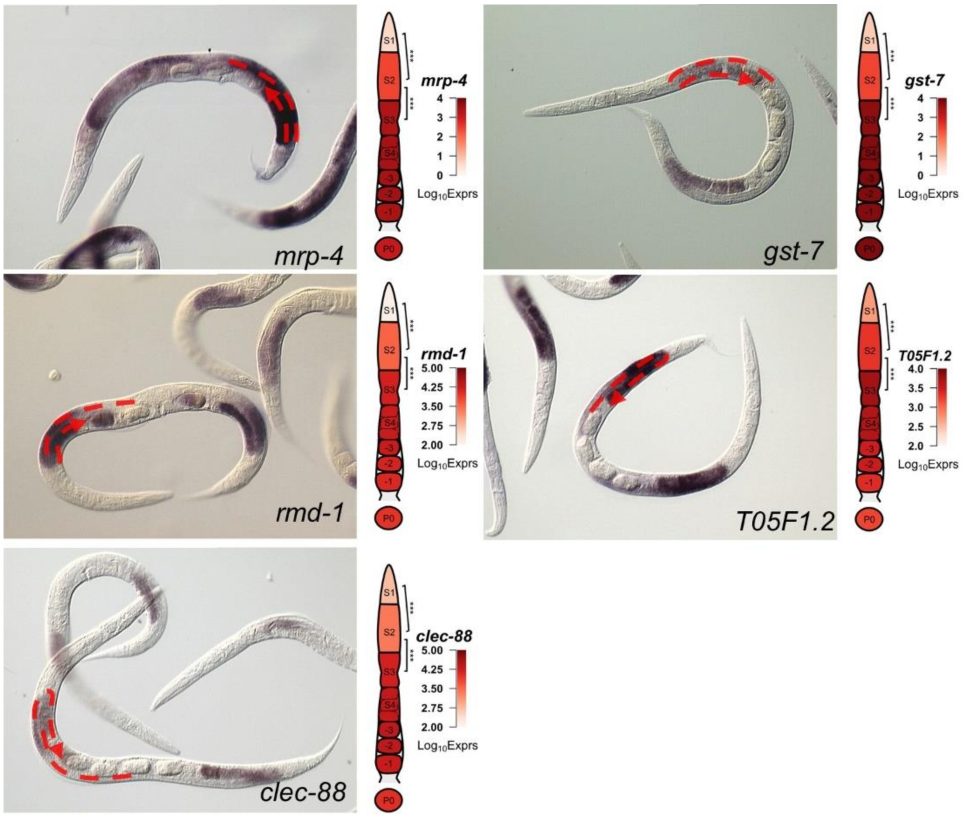

Supplementary Figure S14. ISH images taken from NEXTDB(7) for genes *mrp-4, gst-7, rmd-1, T05F1.2* and *clec-88* in cluster 19, red dashed arrow shows oogenesis path in the gonad. BH p-adj: * < 0.05; ** < 0.01; *** < 0.0001

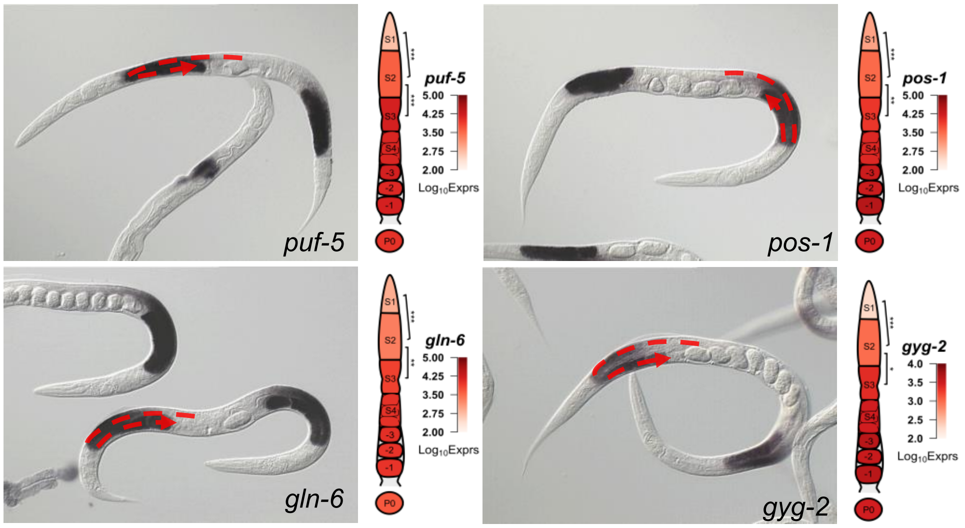

Supplementary Figure S15. ISH images taken from NEXTDB(7) for genes *puf-5, pos-1, gln-6* and *gyg-2* in cluster 20, red dashed arrow shows oogenesis path in the gonad. BH p-adj: * < 0.05; ** < 0.01; *** < 0.0001

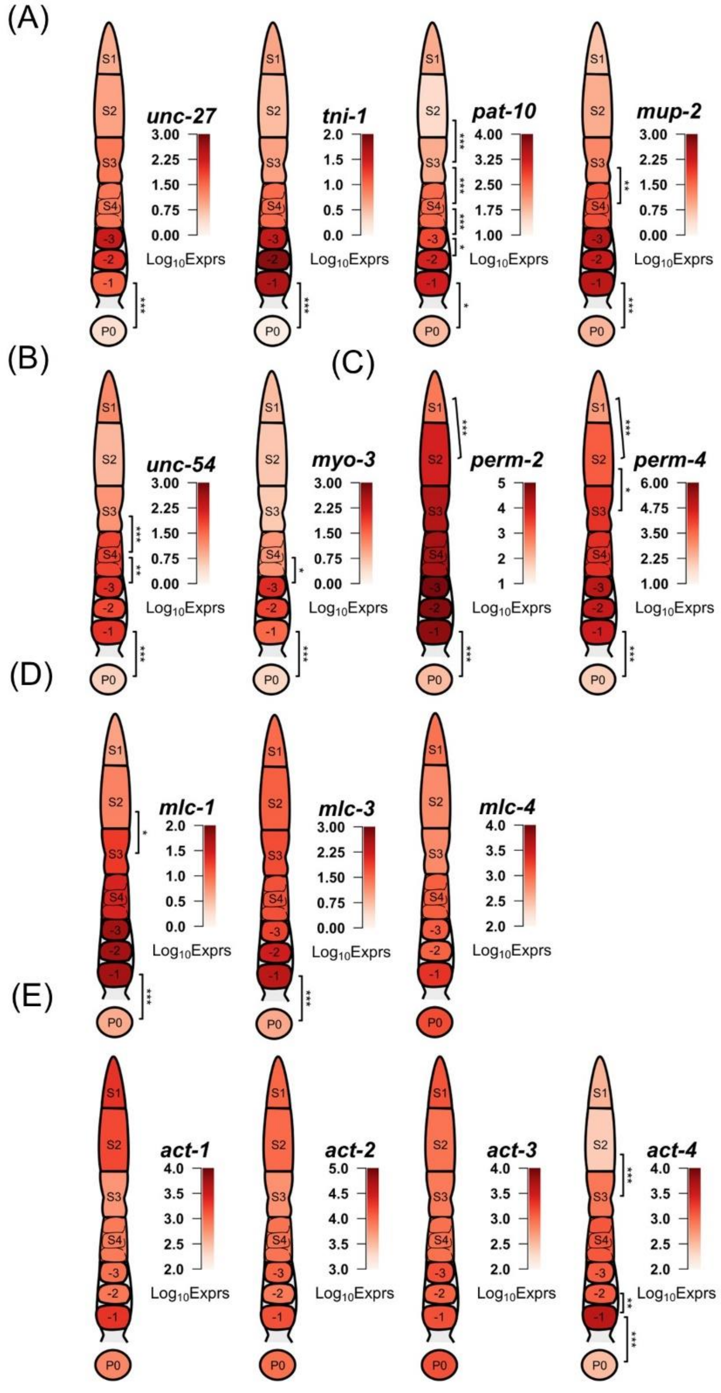

Supplementary Figure S16. A. genes encoding for components of the troponin complex. B. Genes coding for myosin heavy chain proteins (unc-54 and myo-3). C. Genes coding eggshell components perm-2/4. D. Genes coding for four myosin light chains encoding genes. E. Genes coding for four actin encoding genes. BH p-adj: * < 0.05; ** < 0.01; *** < 0.0001

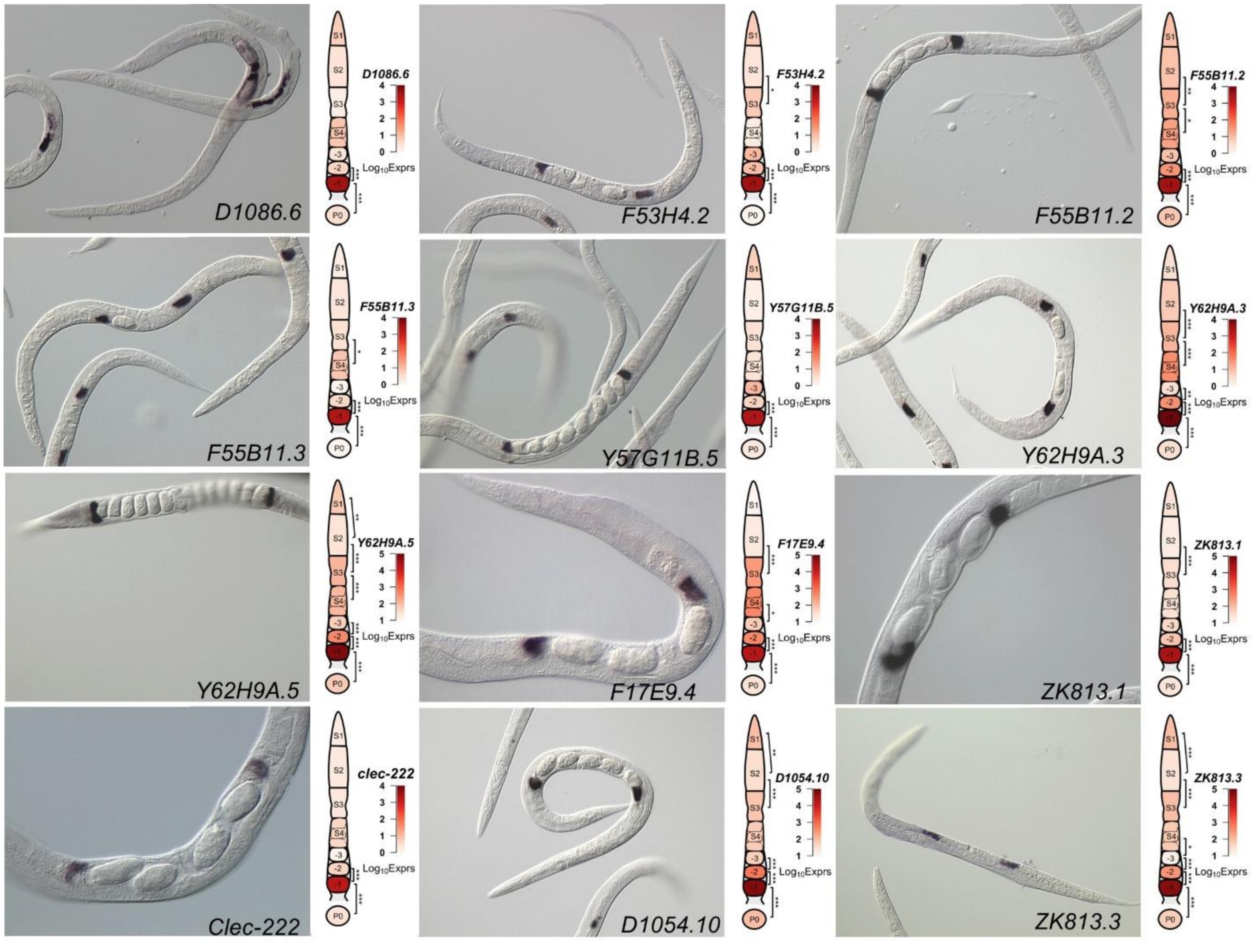

Supplementary Figure S17. NEXTDB(7) in situ imaging of 12 of the 25 putative genes that originate from the spermathecae, related to Figure 5C and Results . BH p-adj: * < 0.05; ** < 0.01; *** < 0.0001

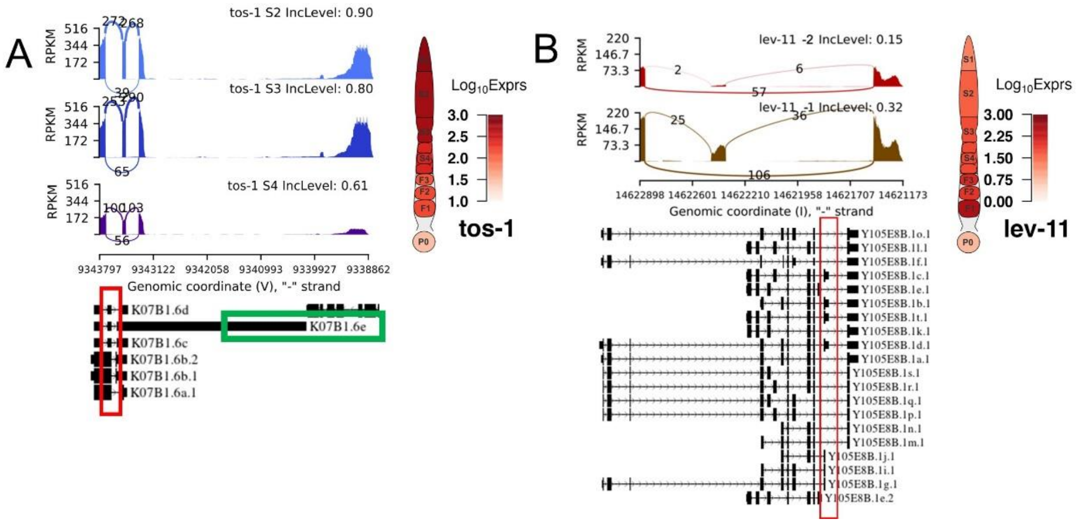

Supplementary Figure S18. A. Differential splicing events of tos-1 between S2 and S3 as well as S3 and S4 stages. B. Differential splicing event of lev-11 between F2 and F1 oocytes. Exact positions of splicing events are shown in the red box, long isoform of tos-1 is shown in green box.

**Our segments**	**Tzur et al, 2018(8)**	**Diag et al, 2018(9)**
**S1**	Segments 1,2	Segments 1,2
**S2**	Segments 3,4,5	Segments 4, 5, 6, 7
**S3**	Segments 6,7	Segment 8
**S4**	Segment 8	Segments 10,11
**F3**	Segment 9	Segment 12
**F2**	Segment 9	Segment 13
**F1**	Segment 10	Segments 14, 15
**P0**	NA	NA

Supplementary Table S1. Collection of mitochondrial, sperm, intestine, stress and rRNA genes used for filtering, related to Methods and Materials. Curated Sperm Genes are obtained from (1, 2), Curated Intestine genes are obtained from (5), Curated stress genes are obtained from (6).

Supplementary Table S2. Correspondence between or gonad stages and that of Diag et al, 2018(9) and Tzur et al, 2018(8), related to Methods and Materials.

Supplementary Table S3. Differential expression results for pairwise comparison between neighboring stages, related to Figure 3C. Stages. Tested column indicates which 2 stages are compared.

Supplementary Table S4. Gene cluster membership for all significant DEGs, related to Figure 4.

Supplementary Table S5. **Significantly enriched gene sets from GSEA analysis on differential expression results of each comparison, related to Results.** Stages. Tested column indicates which 2 stages are compared.

Supplementary Table S6. **121 selected genes for validation with NEXTDB.**

**Putative Genes from Spermathecae**
clec-222, D1054.10, ule-3, D1086.6, F53H4.2, F54F7.3, F55B11.2, F55B11.3, F57C2.4, K07A1.6, Y37D8A.19, Y57G11B.5, Y62H9A.3, Y62H9A.4, Y62H9A.5, ule-5, ZC373.2, E02H9.7, F17E9.4, ZK813.1, ZK813.3, D1086.11, H29C22.1, ZK813.7, F38A5.22

Supplementary Table S7. **25 putative genes that originate from the spermathecae, related to Figure 5C and Results.** Genes with high expression in F1 cells that are significantly upregulated between F2 and F1 and significantly downregulated between F1 and P0.

References

1. Reinke V, Gil IS, Ward S, Kazmer K. Genome-wide germline-enriched and sex-biased expression profiles in Caenorhabditis elegans. Development. 2004;131(2):311-23.
2. Ortiz MA, Noble D, Sorokin EP, Kimble J. A new dataset of spermatogenic vs. oogenic transcriptomes in the nematode Caenorhabditis elegans. G3 (Bethesda). 2014;4(9):1765-72.
3. Harris TW, Arnaboldi V, Cain S, Chan J, Chen WJ, Cho J, et al. WormBase: a modern Model Organism Information Resource. Nucleic Acids Research. 2019.
4. Davis P, Zarowiecki M, Arnaboldi V, Becerra A, Cain S, Chan J, et al. WormBase in 2022-data, processes, and tools for analyzing Caenorhabditis elegans. Genetics. 2022;220(4).
5. Mcghee J. The C. elegans intestine. WormBook. 2007.
6. Brunquell J, Morris S, Lu Y, Cheng F, Westerheide SD. The genome-wide role of HSF-1 in the regulation of gene expression in Caenorhabditis elegans. BMC Genomics. 2016;17(1).
7. Kohara Y. [Systematic analysis of gene expression of the C. elegans genome]. Tanpakushitsu Kakusan Koso. 2001;46(16 Suppl):2425-31.
8. Tzur YB, Winter E, Gao J, Hashimshony T, Yanai I, Colaiacovo MP. Spatiotemporal Gene Expression Analysis of the Caenorhabditis elegans Germline Uncovers a Syncytial Expression Switch. Genetics. 2018;210(2):587-605.
9. Diag A, Schilling M, Klironomos F, Ayoub S, Rajewsky N. Spatiotemporal m(i)RNA Architecture and 3' UTR Regulation in the C. elegans Germline. Dev Cell. 2018;47(6):785-800 e8.
